# Genome-wide mapping of formaldehyde-induced DNA–protein crosslinks reveals unique patterns of formation and transcription-coupled removal in mammalian cells

**DOI:** 10.1093/nar/gkaf720

**Published:** 2025-07-31

**Authors:** Duha Alshareef, Charlie T Nguyen, Kayla N Tucker, Micah D Gearhart, Natalia Y Tretyakova, Colin Campbell

**Affiliations:** Department of Pharmacology, University of Minnesota, Minneapolis, MN 55455, United States; Department of Pharmacology, University of Tabuk, Tabuk, Tabuk 71491, Saudi Arabia; Department of Pharmacology, University of Minnesota, Minneapolis, MN 55455, United States; Department of Pharmacology, University of Minnesota, Minneapolis, MN 55455, United States; Department of Obstetrics, Gynecology and Women’s Health, University of Minnesota, Minneapolis, MN 55455, United States; Department of Medicinal Chemistry, University of Minnesota, Minneapolis, MN 55455, United States; Department of Pharmacology, University of Minnesota, Minneapolis, MN 55455, United States

## Abstract

DNA–protein crosslinks (DPCs) form following exposure to various alkylating agents, including environmental carcinogens, cancer chemotherapeutics, and reactive aldehydes. If not repaired, DPCs can interfere with key biological processes such as transcription and replication and activate programmed cell death. A growing body of evidence implicates nucleotide excision repair (NER), homologous recombination, and other mechanisms in the removal of DPCs. However, the effects of genomic context on DPC formation and removal have not been comprehensively addressed. Using a combination of next-generation sequencing and DPC enrichment via protein precipitation, we show that DPCs induced following exposure to formaldehyde are non-randomly distributed across the human genome, based on chromatin state. The data further show that the efficiency of DPC removal correlates with transcription at loci transcribed by RNA polymerase II. Data presented herein indicate that efficient removal of chromosomal DPCs requires both the Cockayne syndrome group B gene as well as “downstream” TC-NER factor xeroderma pigmentosum group A gene. In contrast, loci transcribed by RNA polymerase I showed no evidence of transcription-coupled DPC removal. Taken together, our results indicate that complex interactions between chromatin organization, transcriptional activity, and numerous DNA repair pathways dictate genomic patterns of DPC formation and removal.

## Graphical Abstract

**Graphical Abstract:**
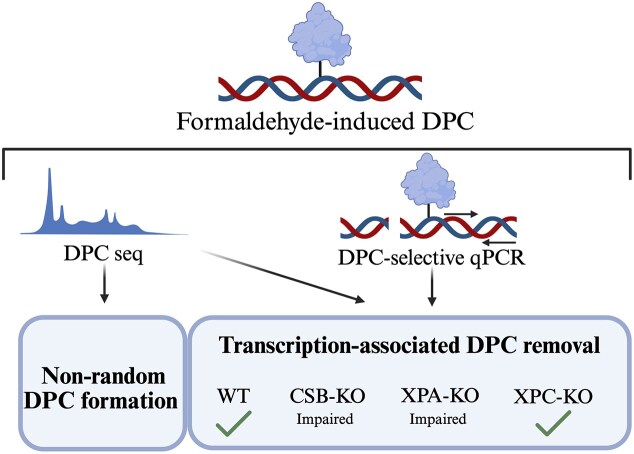

## Introduction

DNA–protein crosslinks (DPCs) are toxic bulky adducts that are created when a covalent bond forms between DNA and cellular proteins. DPCs are generated following exposure to a wide range of endogenous and xenobiotic agents, including endogenous aldehydes and reactive oxygen species, metals, ionizing radiation, and DNA-damaging anticancer drugs such as cisplatin and nitrogen mustards [1–3]. DPCs are also formed endogenously due to enzymatic reactions of topoisomerases and DNA repair enzymes, exposures to cellular metabolites and oxidants, and spontaneous reactions of DNA-interacting proteins with abasic sites on DNA [4, 5]. Because of their bulky size, DPCs interfere with replication and transcription machineries and are associated with genomic instability, mutagenesis, premature aging, and neurodegeneration [3, 6, 7]. Despite their prevalence and toxicity, the repair mechanisms of DPCs are not well understood.

**Figure 1.**
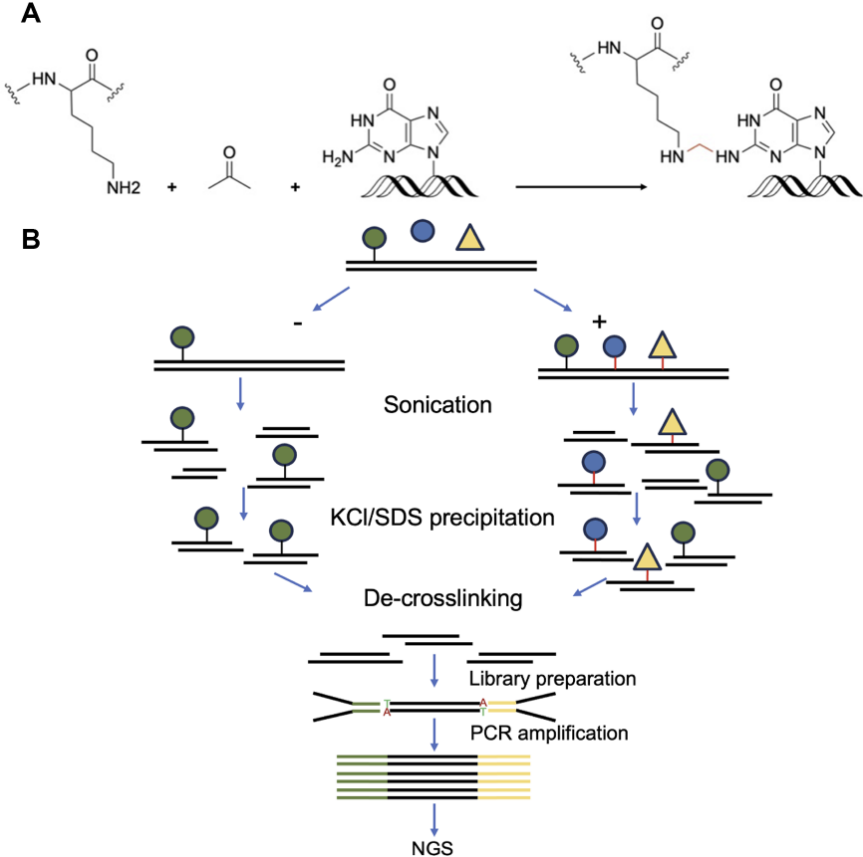
Experimental design of DPC-seq. (**A**) Structure of a model formaldehyde-induced DPC. Shown is a representative DPC formed between a lysine residue and the N² position of deoxyguanosine (N²-dG) in DNA. Formaldehyde can also mediate crosslinks between N²-dG and cysteine residues. (**B**) Schematic of formaldehyde-induced DPC-seq protocol. Circles and triangles depict the heterogenous mixture of proteins crosslinked to DNA spontaneously with no treatment (−, green colored circles) or upon formaldehyde treatment (+, blue colored circles and triangle).

Formaldehyde is a potent DPC-inducing agent and a classified carcinogen [8]. It occurs both endogenously as a byproduct of normal metabolic processes and oxidative stress, and exogenously as an environmental and occupational toxin [9, 10]. The electrophilic aldehyde group in formaldehyde reacts with nucleophilic amine groups in DNA and proteins, leading to the formation of DPCs. A model DPC is depicted in Fig. 1A. Formaldehyde-induced DPCs have been extensively studied in an effort to understand their cytotoxicity, chemical structure, and repair mechanisms [8–11]. Furthermore, the proteins participating in DPC formation have been characterized using mass spectrometry-based proteomics to identify the nature of proteins that become crosslinked to DNA [4, 12–16].

Depending on their genomic location, DNA lesions may have different biological effects due to their interference with gene expression and replication. Therefore, there is a growing interest in mapping the formation and repair of different DNA damage across the human genome. These unbiased approaches provide insights into factors influencing DNA adduct formation, recognition, and repair mechanisms. For example, high-sensitivity damage sequencing (HS-Damage-seq) and excision repair sequencing (XR-seq) were developed to map UV-induced DNA damage formation and repair, respectively, at a single-nucleotide resolution in the human genome [17–19]. These sequencing studies collectively highlight the uniform distribution of UV-induced photoproducts across the human genome, but their preferential removal from actively transcribed genes in a lesion-dependent manner. Damage-seq and XR-seq were also used to map cisplatin-induced DNA–DNA crosslinks, where similarly the damage formation was uniform but the repair was heterogenous [20].

Other investigators have utilized analogous approaches to map genomic loci where specific proteins localize at sites of DNA damage. One such approach, referred to as protein-associated DNA damage sequencing (PADD-seq), used chromatin immunoprecipitation to localize RNA polymerase II-DNA interactions at ultraviolet radiation-induced DNA damage and histone H3 acetylated on lysine 9 at sites of cisplatin-induced DNA damage [21]. Similarly, covalent crosslinking of DNA glycosylase endonuclease III-like protein 1 (NTHL1) was used to generate a genome-wide map of spontaneously formed thymidine glycol lesions [22]. Both of these latter techniques rely on antibodies to specifically recognize individual DNA-crosslinked proteins. However, there is considerable interest in comprehensively identifying all genomic loci where cellular proteins become covalently crosslinked to DNA. This is an important objective because xenobiotic-induced DPCs are distinct from other types of DNA damage that have been subjected to genome-wide analysis in three main ways: (i) they exhibit greater chemical complexity and diversity, (ii) they are dependent on close interactions between genomic DNA and nuclear proteins, and (iii) due to their complexity and heterogeneity, they are subject to a spectrum of repair pathways. Cellular proteins interact dynamically with DNA, which is spatially organized into non-random positions and higher-order structures [23, 24]. In addition, considerable uncertainty exists as to the extent to which genomic organization and identified genomic domains influence DPC formation and removal.

To address these questions, we pursued a comprehensive unbiased approach combining protein precipitation [25, 26] with next-generation sequencing and qPCR to determine the formation and removal patterns of formaldehyde-induced DPCs across the human genome. While our experiments were in progress, a series of papers were published that similarly utilized unbiased approaches to examine the dynamics of formaldehyde-induced DPCs across the human genome [27–29]. These studies showed that a transcription-coupled repair mechanism resolved formaldehyde-induced chromosomal DPCs. The data presented herein extend these recently published findings and provide additional insight into the molecular biology of chromosomal DPC repair. Importantly, our data convincingly demonstrate that DPCs are subject to transcription-coupled nucleotide excision repair (TC-NER) that requires the Cockayne syndrome group B (CSB) protein as well as downstream factor xeroderma pigmentosum group A (XPA). Our data further indicate that ribosomal DPCs are not similarly subject to TC-NER repair.

The findings offer valuable insights into the cellular response and recognition mechanisms involved in combating DNA damage induced by xenobiotic agents. Moreover, this technique serves as a tool for investigating the genome-wide formation and removal dynamics of DPCs induced by chemotherapeutic agents such as cisplatin and mechlorethamine, potentially improving chemotherapy management.

## Materials and methods

### Reagents and materials

Sixteen percent formaldehyde ampules were purchased from Thermo Scientific (REF 28906). One milliliter milliTUBE (part number: 520135) was purchased from Covaris. QIAquick PCR purification kit (REF 28106) and MinElute Gel extraction kit (REF 28604) were purchased from Qiagen. Qubit dsDNA High Sensitivity Assay kit (Q32851) and E-Gel EX 2% Agarose were purchased from Invitrogen (REF G401002). NEBNext Ultra II DNA prep kit was purchased from New England Biolabs (E7645S). Indexed primers were either purchased from NEB (NEBNext Multiplex Oligos for Illumina, Index Primers, set 1) or custom designed and ordered from Integrated DNA Technologies. Eagle’s Minimum Essential Media (MEM) and fetal bovine serum were purchased from Gibco. All other chemicals were obtained from Sigma–Aldrich. Xeroderma pigmentosum (XP) group A (XPA) mouse monoclonal antibody (ab65963) and Cockayne syndrome (CS) group B (CSB) rabbit polyclonal antibody (ab96089) were purchased from abcam. XPC rabbit polyclonal antibody was purchased from Cell Signaling (12701T). β-actin mouse monoclonal antibody (E0615) and goat anti-rabbit HRP-conjugated secondary antibody (G1715) were purchased from Santa Cruz Biotechnology. Goat anti-mouse HRP-conjugated secondary antibody was purchased from Invitrogen (626520). Luna Cell Ready One-Step RT-qPCR kit was purchased from New England Biolabs (E3032). pcDNA4-Flag-XPA plasmid was purchased from Addgene (22895). Stocks of zeocin were purchased from Invitrogen (R25001). Reverse transcription quantitative polymerase chain reaction (RT-qPCR) primers were custom designed and ordered from Integrated DNA Technologies (Supplementary Table S1).

### Cell line

Human fibrosarcoma cell line (HT1080) was purchased from ATCC (CCL-121) and HT1080-XPA-KO cells were ordered from Synthego. HT1080-XPC-KO and HT1080-CSB-KO cells were purchased from EditCo as a knockout cell pool. Cells were cultured in MEM supplemented with 9% fetal bovine serum. Cells were maintained in 5% CO_2_ humidified incubator at 37°C.

Single-cell isolation from XPA-KO, XPC-KO, and CSB-KO HT1080 cell pools was done using limiting dilution followed by clonal expansion. The knockout clones were confirmed via genotyping for XPA-KO cells using XPA sequencing primer pair (Supplementary Fig. S8; Supplementary Table S1) and western blotting for all cell lines (Supplementary Fig. S9).

XPA-KO cells were stably complemented (XPA-KO-corr) with the XPA gene using the pcDNA4-Flag-XPA plasmid. Briefly, 5 μg of pcDNA4-Flag-XPA plasmid was mixed with lipofectamine in Opti-MEM media and incubated at room temperature for 15 min. The transfection mixture was then added to XPA-KO cells in a 24-well plate after aspirating the previous media, followed by incubation at 37°C for 24 h. The next day, the media was replaced with complete media, and cells were incubated at 37°C for an additional 48–72 h. Cells were then split at a 1:3 ratio into zeocin selection media (70 μg/mL) and maintained with fresh selection media every 3–4 days until resistant colonies formed. Stably transfected colonies were isolated, expanded, and confirmed for XPA expression using western blotting (Supplementary Fig. S9A).

### Western blot

Nuclear extracts were prepared from WT, XPA-KO, XPA-KO-corr, XPC-KO, and CSB-KO HT1080 cells as previously described [30]. Protein concentration was determined using the bicinchoninic acid (BCA) assay, and 20 μg of protein was separated on a Bolt Bis-Tris Plus 4–12% SDS–PAGE (sodium dodecyl sulfate–polyacrylamide gel electrophoresis gel). After electrophoresis, proteins were transferred onto a nitrocellulose membrane and blocked with 2.5% non-fat milk at room temperature for 1 h. Membranes were incubated overnight at 4°C with primary antibodies against XPA, XPC, CSB (1:2000 dilution), or β-actin (1:1000 dilution). After multiple washes with Tris-buffered saline containing 0.1% Tween 20, membranes were incubated with HRP-conjugated secondary antibodies (1:10 000 dilution), then blots were developed using Pierce ECL western blotting substrate (32209, Thermo Scientific) and imaged using Bio-Rad ChemiDoc imaging system.

### Cell treatments

Cells were treated with 400 μM formaldehyde for 2 h or 50 μM mechlorethamine for 1 h in serum-free medium supplemented with 25 mM HEPES. Cells were treated with 1 μM dexamethasone overnight in complete media.

### The development of the formaldehyde-induced DPC-seq procedure

Formaldehyde-induced DPC-seq was developed from existing methodologies [25, 26] that selectively enrich for protein-linked DNA combined with ultrasonication to generate fragments with a specific size range optimized for sequencing (Fig. 1B).

Approximately 1.2 million HT1080 cells were incubated in the presence or absence of 400 μM formaldehyde in serum-free MEM media for 2 h. This treatment regimen is similar to those utilized previously by other investigators who examined the dynamics of DPC removal from transcribed loci [27–29]. Van Sluis *et al.* [29] determined that a 30-min exposure to 1 mM formaldehyde primarily reduced transcription elongation rather than blocked *de novo* transcription initiation, while Oka *et al.* [27] showed that inhibition of transcription with triptolide and 5,6-dichloro-1-beta-D-ribofuranosylbenzimidazol significantly reduced DPC removal, indicating that transcription elongation is required for efficient DPC resolution following a 1 h exposure of cells to 600 μM formaldehyde. Following treatment, media was removed, and cells were immediately collected (T) or allowed to recover for 5 h (R) in serum-supplemented MEM media. Cells were then subjected to the ARK, advanced recovery of potassium-SDS precipitates, assay, which is a modified KCl/SDS precipitation methodology [25, 26]. Analysis of the enriched materials by agarose gel electrophoresis indicates a more than six-fold induction of DPCs in formaldehyde-treated cells compared to untreated control (Supplementary Fig. S1).

To generate genome-wide maps of formation and removal of formaldehyde-induced DPCs, the ARK-KCl/SDS precipitation procedure was modified to include an ultrasonication step. DNA fragments in the 200–500 bp size range were generated using material from untreated control samples (C), as well as from samples collected immediately following initial formaldehyde treatment (T) and 5-h recovery (R). Briefly, media was gently aspirated, and cells were lysed with MB buffer [5.6 M guanidine thiocyanate, 10 mM Tris–HCl (pH 6.5), 20 mM ethylenediaminetetraacetic acid (EDTA), 4% Triton X-100, 1% Sarkosyl, and 1% DTT] that was prewarmed at 55°C. After scraping, the viscous lysate was passed through a 1 mL pipette tip six times, then transferred into a 1.5 mL Eppendorf tube. The lysate was further sheared by passing it through a 21-gauge needle six times, then an equal volume of pre-chilled 100% ethanol was added to precipitate free and protein-linked DNA. Following sedimentation at 21 000 × *g* at 4°C for 20 min, the supernatant was carefully removed, and the pellet was washed with an ethanol buffer [20 mM Tris–HCl (pH 6.5), 150 mM NaCl, and 50% ethanol]. The wash buffer was carefully aspirated and the remaining sedimented material was stored overnight at −80°C with no air-drying. The next day, ethanol-precipitated DNA was resuspended in 1 mL 1% SDS buffer containing 20 mM Tris–HCl, pH 7.4, sheared by passing through a 25-gauge needle six times, transferred to a 1 mL Covaris milliTUBE, and subjected to adaptive focused acoustics ultrasonication for 12–14 min in a Covaris S-220 instrument following the manufacturer’s protocol. Sonication time was preoptimized based on the number of cells and treatment conditions, in order to generate DNA fragments in the 200–500 bp size range. Following ultrasonication, KCl/SDS precipitation was performed as previously described with the following modifications. Briefly, 1M KCl and 1M Tris–HCl, pH 7.4, were added to a final concentration of 100 mM and 20 mM, respectively, to precipitate protein-linked DNA. Samples were chilled on ice for 5 min, then sedimented at 21 000 × *g* at 4°C for 5 min. The precipitated protein-linked DNA was then washed with 1.5 mL of 100 mM KCl buffer containing 20 mM Tris–HCl, pH 7.4, heated at 55°C, chilled on ice, then centrifuged again. This wash step was repeated, and the final pellet containing protein-linked DNA was resuspended in 100 mM KCl buffer containing 20 mM Tris–HCl, pH 7.4, 10 mM EDTA, and 0.2 mg/mL proteinase K, and incubated overnight at 55°C to digest DPCs and reverse formaldehyde-DNA crosslinks. The next day, samples were chilled on ice for 5 min, and supernatant was carefully collected after centrifugation (21 000 × *g*, 4°C for 10 min). Samples were then purified using a Qiagen PCR purification column, and amount of recovered DNA was determined with the Qubit dsDNA High Sensitivity Assay Kit, following the manufacturer’s guidelines (Supplementary Fig. S2). Finally, equal amounts of recovered DNA from each group were used to prepare DNA libraries for next-generation sequencing as detailed below.

### DNA library preparation

DNA libraries were prepared using the NEBNext Ultra II DNA Prep Kit for Illumina following the manufacturers protocol. Material obtained as described earlier was resolved using the E-Gel EX 2% agarose gel ran at 35 V for 1.5 h. Size-selected libraries, containing DNA in the 200–500 bp range, were purified using the MinElute Gel extraction kit and quantified as described earlier. For library quality control, libraries were diluted to 1 pM and amplified with kapa primers (Supplementary Table S1), where similar Ct values were expected. Then, libraries were pooled at equimolar ratios. Electrophoresis was performed using a TapeStation apparatus (Agilent) to determine the size distribution of pooled libraries (Supplementary Fig. S3).

### Sequencing data analysis

High-throughput sequencing was performed on prepared libraries (Azenta/Genewiz, South Plainfield, NJ) using HiSeq platforms with 150 paired-ends at ∼30 million reads per library. Adaptor sequences were removed with Trim Galore (v 0.6.6). Trimmed reads were then mapped onto the human genome (hg38) using the default settings of BWA MEM (v 0.7.17). Exclusion regions for hg38 were downloaded from (https://github.com/Boyle-Lab/Blacklist/tree/master/lists) and were removed with Bedtools (v 2.29.2) along with the mitochondrial chromosome. PCR duplicates were removed with Picard MarkDuplicates (v 2.25.6). Reads were quality filtered (-bq 55), sorted, and indexed using SAMtools (v 1.9).

MACS2 (v 2.2.8) was used to identify peaks in formaldehyde-treated samples compared to untreated controls using the following parameter (--nomodel --nolambda --broad). For untreated control samples, no control file was used to identify the peaks.

OverlapEnrichment command from ChromHMM was used to calculate the fold enrichment of each chromatin state to C, T, and R peak lists generated by MACS2. The fold enrichment is computed as (number of bases in the state and peak list/number of bases in the state)/(number of bases in the peak list/number of bases in the genome).

All samples were normalized as counts per million mapped reads (CPM) using deepTools (v 3.5.2) command bamCoverage with the following parameters (--binSize 10 --normalizeUsing CPM --ignoreForNormalization chrM --extendReads). Screenshots of normalized reads were plotted with Integrative Genomics Viewer (v2.13.1).

To generate gene expression quintiles, Fragments Per Kilobase of transcript per Million mapped reads (FPKM) of HT1080 polyA plus RNA-seq [31] (accession number ENCFF691EGO) was used to divide gene expression into four quintiles (Q1–Q4) with a threshold of FPKM = 0.1 for Q1. Q0 was generated by sampling genes that have FPKM = 0. Heatmaps were plotted using EnrichedHeatmap (v 3.17).

For correlation heatmap, 50 000 peaks with a width of 1660 ± 2600 were sampled from 662 508 merged peaks to calculate Spearman’s correlation between these initial treatments and ATAC-seq, RNA polymerase II, H3K4me3, H3K27ac, H3K27me3, and H3K9me3 signals of HT1080 cells (see accession numbers below).

### rDNA data analysis

To map DPC reads to rDNA, the canonical sequence of the human rDNA was used to rebuild the reference genome for ribosomal genes as described previously [32]. Briefly, the 13 357-long human 45S pre-ribosomal N5 sequence was downloaded from NCBI (RNA45SN5, accession number NR_046235), and fasta files were generated. We also included 100 non-transcribed genes and 100 highly transcribed genes from Q0 and Q4 quintiles as positive and negative controls, respectively. Since the first 50 bp of the 150-PE reads are less susceptible to sequencing errors, reads were trimmed to only include the first 50 bp from the 5′ end after trimming adaptor sequences using Trim galore (v 0.6.6) as follows (--hardtrim5 50 file.R1.fq.gz). Fifty base pair single-end (SE) reads were then mapped to the reconstructed rDNA genome. Mapped reads were filtered with R (v 4.2.2) to remove any mismatches, then the total number of filtered reads was calculated for Q0, Q4, and rDNA genes at initial treatment (T samples) and after a 5-h recovery period (R samples) (Supplementary Table S3). To calculate the relative removal efficiency (RRE), total number of filtered reads at T was divided by total number of filtered reads at R, then the average of the three replicates was plotted as a bar graph.

As an alternative approach, we mapped the 50 bp SE reads to the hg38 genome, extracted reads that map to 3429 and 4353 genes from Q0 and Q4, respectively, and filtered out reads with mismatches using R (v 4.2.2). Then the total number of reads mapping to each group was calculated using summarizeOverlaps (GenomicRanges, v 3.18).

### Reprogramming transcription with dexamethasone

Briefly, 50% confluent HT1080 cells were pre-treated with 1 μM dexamethasone overnight in complete MEM media. The next day, cells were treated with 400 μM formaldehyde for 2 h, then collected immediately following treatment, or following a 5-h formaldehyde-free recovery period as described earlier. During the 5-h recovery, media was supplemented with 1 μM dexamethasone. Recovered DNA was sheared with adaptive focused acoustics using Covaris S-220 for 12–14 min following the manufacturer’s protocol, and KCl/SDS precipitation was performed as described earlier. The final pellet containing protein-linked DNA was then resuspended in a 100 mM KCl buffer containing 20 mM Tris–HCl, pH 7.4, 10 mM EDTA, and 0.2 mg/mL proteinase K, and incubated overnight at 55°C to digest DPCs and reverse formaldehyde-DNA crosslinks. Protein-linked DNA was then purified as described earlier and subjected to qPCR with a primer set delimiting a 178 bp amplicon specific for the interleukin 1-beta (IL1B) gene (Supplementary Table S1). The IL1B gene was selected because it displayed high transcription activity in normal growth media that was significantly repressed upon dexamethasone exposure [33]. The percent of DPC removal from this locus was calculated using the following formula: [1 − 0.5^(delta Ct)] × 100, where delta Ct = (Ct of samples harvested from cells permitted to recover for 5 h post-formaldehyde treatment) − (Ct of samples harvested from cells immediately following formaldehyde treatment). Experiments where the delta Ct value was negative were discarded.

### RT-qPCR

RT-qPCR was performed using the Luna Cell Ready One-Step RT-qPCR kit according to the manufacturer’s protocol on cells pre-treated overnight with or without 1 μM dexamethasone. Primer pairs targeting IL1B (175 bp amplicon) and GAPDH (170 bp amplicon) complementary DNA (cDNA) were used. Gene expression was normalized to GAPDH as the housekeeping gene by calculating ΔCt, and the fold change in IL1B expression was determined using the 2^−ΔΔCt method.

### DPC-selective qPCR

Wild-type (WT), XPA-KO, XPA-KO-corr, XPC-KO, or CSB-KO HT1080 cells were treated with formaldehyde and drugs as described in the text, and protein-linked DNA was recovered as described earlier. To examine DPC removal at selected loci, qPCR was performed with primer pairs specific of a single locus (IL1B) or a set of three primer pairs selective for the rDNA repetitive genes (Supplementary Table S1). The percent DPC removal was calculated as described earlier. Experiments where the delta Ct value was negative were discarded.

### ENCODE data

HT1080-specific ChromHMM segmentation (accession number ENCFF461DZD), HT1080 polyA plus RNA-seq (accession number ENCFF691EGO), HT1080-H3K4me3 (accession number ENCFF688MFO), HT1080-H3K27ac (accession number ENCFF090WXN), HT1080-H3K27me3 (accession number ENCFF633FBI), HT1080-H3K9me3 (accession number ENCFF408PEC), HT1080-ATAC-seq (accession number ENCFF827ACX), and HT1080-RNAPolII ChIP-seq (accession number ENCFF544VBI) data were downloaded from ENCODE portal. RNA45SN5 canonical sequence (accession number NR_046235) was downloaded from NCBI.

### Statistical analysis

**Figure 2.**
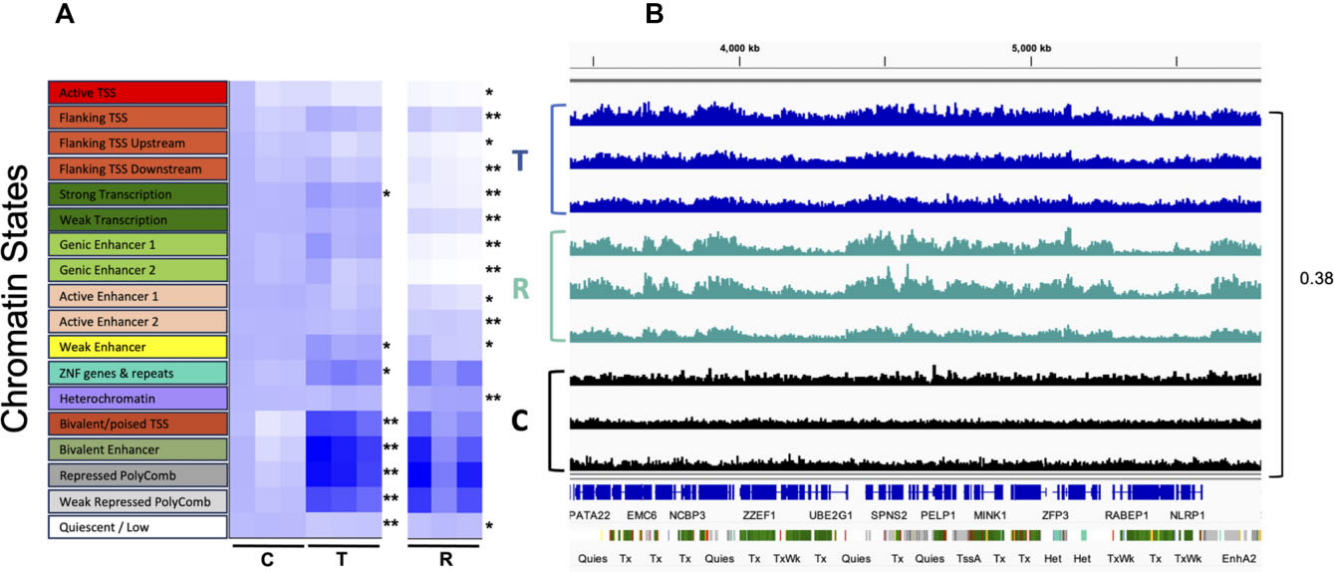
Formation patterns and regioselective removal of formaldehyde-induced DPCs. (**A**) Fold enrichment of DPCs across 18 chromatin states for untreated control (C), immediately after exposure to 400 μM formaldehyde for 2 h (T), and following a 5-h recovery period in drug-free media (R). Darker color corresponds to a higher fold-enrichment with one color scale for the entire map. Summary of *P* values comparing C and T (left) and T and R (right) within each chromatin state is indicated (*P < .05, **P < .01, N = 3). (**B**) High-resolution view depicting relative abundance of DPCs present on a 2000 kb region of chromosome 17 (17:3427029–5586518) in untreated (**C**), 2-h formaldehyde-treated (T), and treated cells permitted to recover in drug-free media (R). Genomic features and ChromHMM tracks are indicated in blue and green, respectively (see the “Materials and methods” section for details). The green-colored regions in ChromHMM track indicate actively transcribed genes. The *y*-axis represents normalized coverage, with group auto-scaling applied to all tracks (maximum value set to 0.38). Data from three independent experiments.

**Figure 3.**
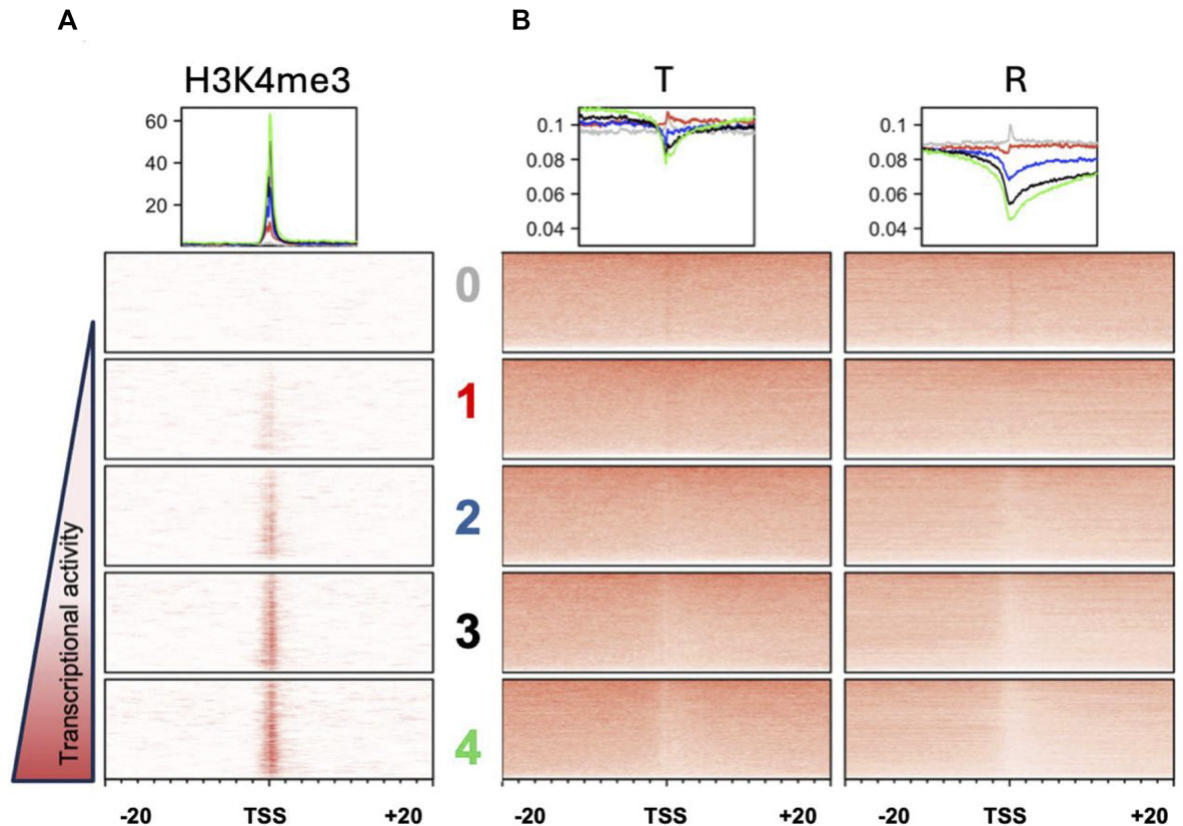
DPC removal is associated with transcription. (**A**) Heatmap representing the intensity of H3K4me3 signals across five quintiles of genes in human HT1080 cells. (**B**) DPC-seq data for cells immediately following a 2-h exposure to formaldehyde (T), and in formaldehyde-treated cells permitted to recover in drug-free media for 5 h (R). The maps are centered at the transcription start site (TSS) across a 40-kb window. The box above the heatmap depicts the signal intensity for each of the five gene sets, Q0 (gray), Q1 (red), Q2 (blue), Q3 (black), and Q4 (green), described in the text. Data from three independent experiments.

**Figure 4.**
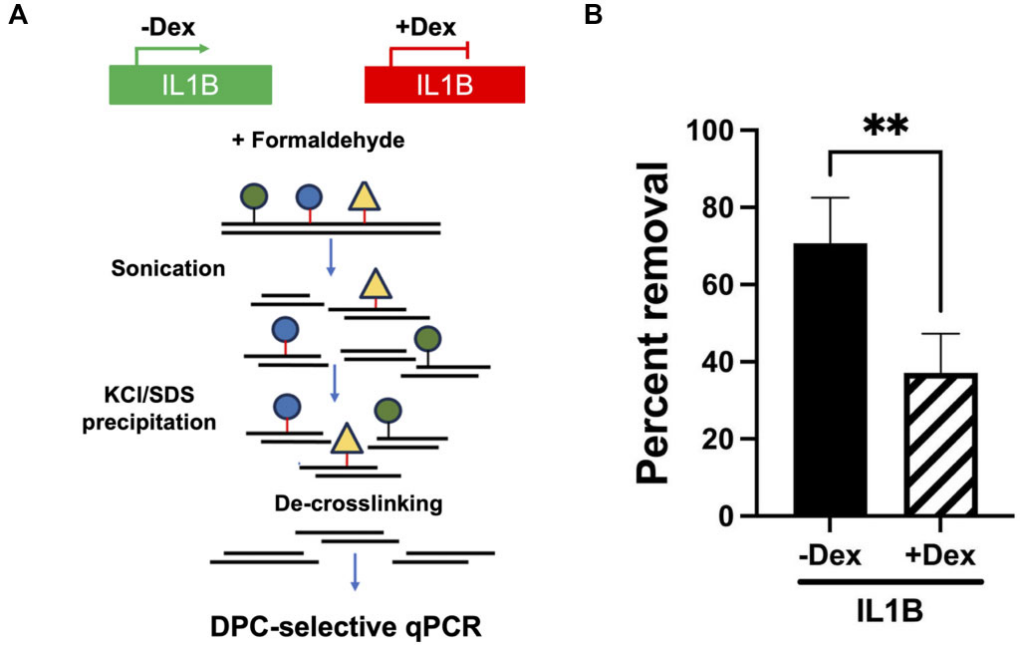
Reprogramming transcription alters DPC removal efficiency. (**A**) Representation of experimental design. HT1080 cells were incubated in the presence or absence of 1 μM dexamethasone overnight and subsequently treated with 400 μM formaldehyde for 2 h. Samples were recovered immediately following formaldehyde exposure or after a 5-h drug-free recovery period, and DPC-selective qPCR was performed (see the “Materials and methods” section for details). (**B**). Formaldehyde-induced DPC selective qPCR was performed on cells that had been pre-incubated in the absence (−Dex) or presence (+Dex) of dexamethasone using a primer pair specific for the IL1B locus. See the “Materials and methods” section and Supplementary Table S1. (*P= .004, N = 5). Error bars represent SEM.

**Figure 5.**
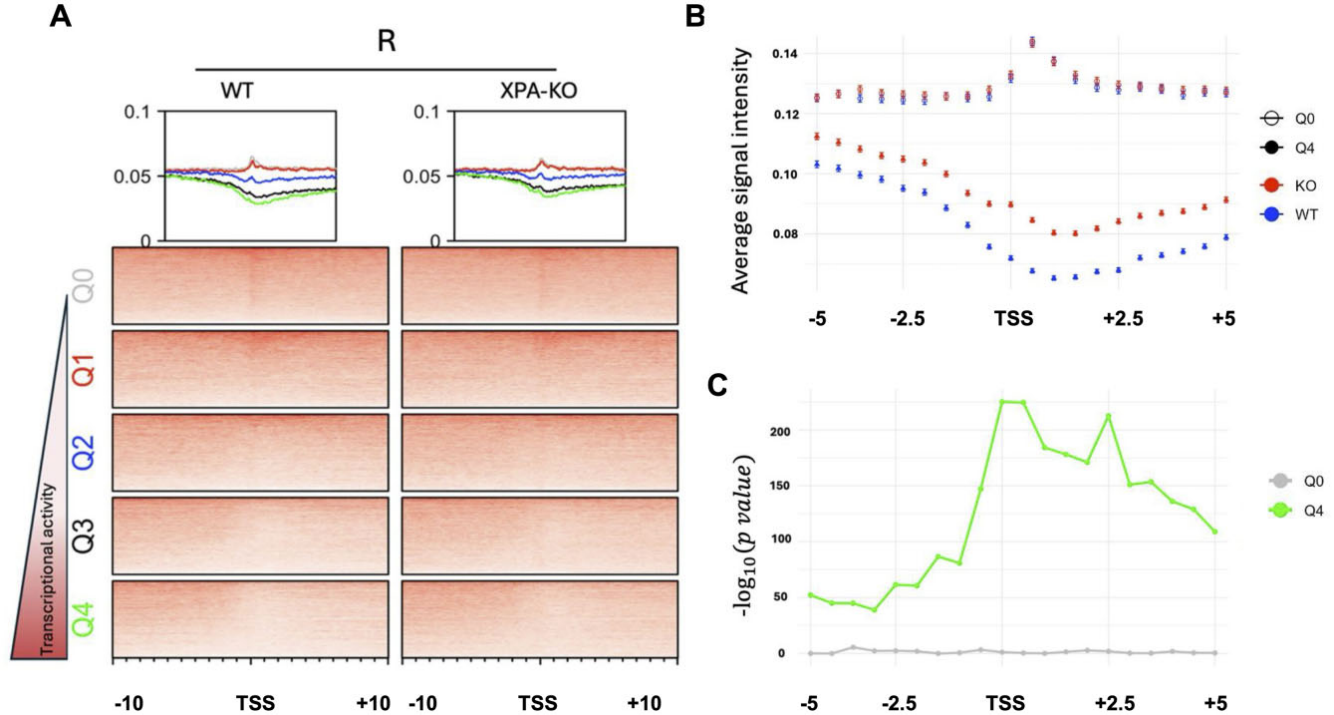
Transcription-coupled DPC removal is impaired in NER-deficient cells. (**A**) Heatmap representing the signal intensity of DPC-seq analysis on unmodified HT1080 cells WT and on a clone of HT1080 cells in which the XPA gene was inactivated (XPA-KO, see the “Materials and methods” section for details) exposed to 400 μM formaldehyde and permitted to recover for 5 h. This analysis was performed across five quintiles composed of genes with differing levels of transcriptional activity (see legend to Fig. 4B), TSS. (**B**) Average signal intensity of DPC-seq signals across a 10 kb region centered on the transcriptional start site for the Q0 (open circles) and Q4 gene sets (filled circles) for WT (blue) and XPA-KO (red) cells. (**C**) −${\log _{10}}p$ values comparing WT versus XPA KO cells across the Q0 (gray) and Q4 (green) gene sets. Data from two independent experiments.

**Figure 6.**
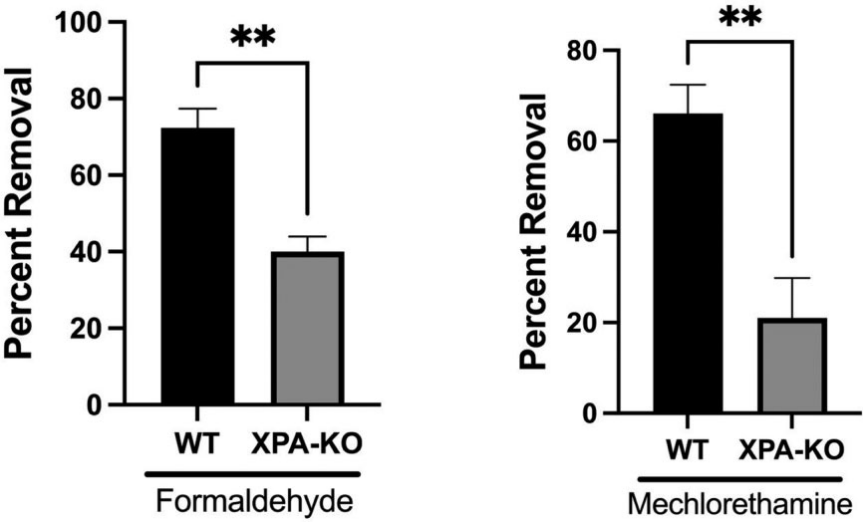
Removal of DPCs at the IL1B locus is partially deficient in XPA-KO cells. DPC selective qPCR was performed using IL1B-specific primers (see the “Materials and methods” section for details) on material recovered from HT1080 cells WT and XPA-KO cells that had been treated with formaldehyde (400 μM, 2 h) or mechlorethamine (50 μM, 1 h) and permitted to recover for 5 h in drug-free media. **P= .002, N = 5 (formaldehyde); **P= .001, N = 3 (mechlorethamine). Error bars represent SEM.

**Figure 7.**
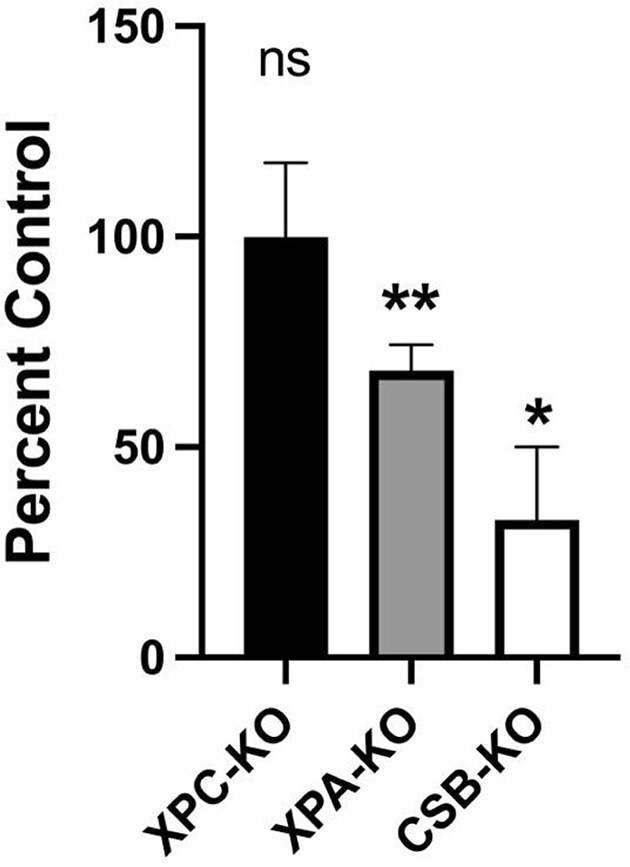
Removal of DPCs at the IL1B locus in different cell lines. DPC selective qPCR was performed using IL1B-specific primers on material recovered from XPC-KO, XPA-KO, and CSB-KO HT1080 cells that had been treated with formaldehyde (400 μM, 2 h) and permitted to recover for 5 h in drug-free media. Data plotted as a percent control of WT, XPA-KO-corrected, and WT, respectively. *P= .04, **P= .01, n.s.; not significant, N = 5 (XPC-KO and XPA-KO), N = 3 (CSB-KO). Error bars represent SEM.

**Figure 8.**
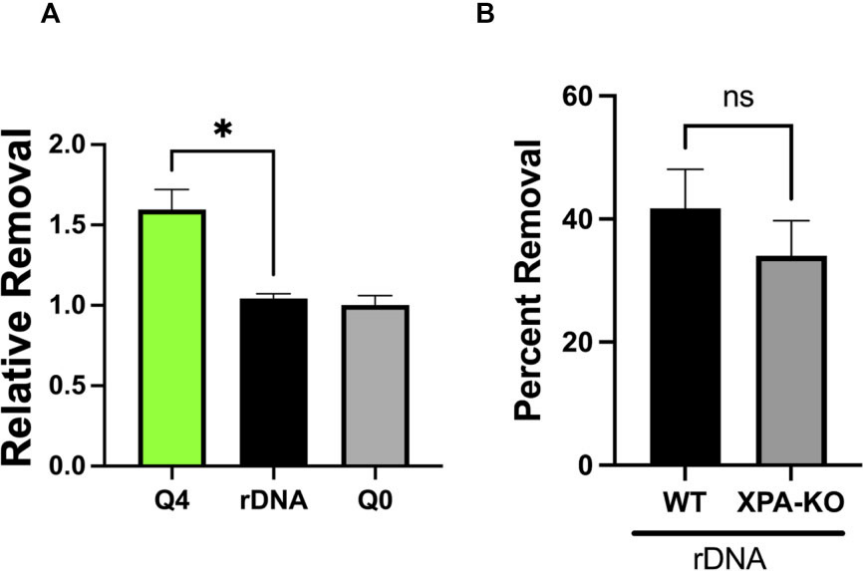
DPC removal from rDNA. (**A**) HT1080 cells were treated with 400 μM formaldehyde for 2 h and permitted to recover in drug-free media for 5 h, and relative efficiency of formaldehyde-induced DPC removal at the rDNA Q0 and Q4 loci was determined (see text and “Materials and methods” section for details). *P= .02, N = 3. (**B**) WT and XPA-KO HT1080 cells were treated with 400 μM formaldehyde for 2 h, and material was harvested immediately or after a 5-h drug-free recovery period. DPC selective qPCR was performed using three sets of primer pairs specific for the 18S, 28S, and 5S rDNA genes (Supplementary Table S1), and the percent removal of formaldehyde-induced DPCs was calculated as described in the “Materials and methods” section. n.s.; not significant, N = 9. Error bars represent SEM.

Results depicting the distribution of spontaneous DPCs and the formation and removal of formaldehyde-induced DPCs represent the average of three independent experiments (Figs 2 and 3, and Supplementary Figs S5 and S6). Results from DPC-selective qPCR-based determination of the effect of transcriptional reprogramming with dexamethasone on removal of DPCs from the IL1B locus represent the average of five independent experiments (Fig. 4). Results depicting the relationship between transcriptional activity and the initial formation and removal of formaldehyde-induced DPCs in WT and XPA-KO cells represent the average of two independent experiments (Fig. 5, and Supplementary Figs S10 and S11). Results from DPC-selective qPCR-based determination of the effect of inactivation of the XPA gene on removal of DPCs from the IL1B locus represent the average of five (Fig. 6, left panel) or three (Fig. 6, right panel) independent experiments. Similarly, results from DPC-selective qPCR on XPC-KO, XPA-KO-corr, and CSB-KO cells represent the average of five, five, and three independent experiments, respectively (Fig. 7). Results depicting the relative efficiency with which formaldehyde-induced DPCs were removed from the rDNA locus (relative to other genomic loci) represent the average of three independent experiments (Fig. 8A and Supplementary Fig. S13). Results examining the removal efficiency of DPCs from the rDNA loci in WT and XPA-KO cells represent the average of nine independent experiments (Fig. 8B). Results depicting selective enrichment of formaldehyde-induced DPCs with the ARK assay are based on the average of three independent experiments (Supplementary Fig. S1). Results depicting the amount of precipitated DNA after KCl/SDS treatment represent the average of three independent experiments (Supplementary Fig. S2). *P* values for Figs 2A and 4–8, and Supplementary Figs S1, S2, and S13 were calculated using paired one-tailed *t*-test. All bar graph data are plotted as mean values with error bars representing standard error of the mean (SEM).

## Results

### Formaldehyde-induced DPCs are non-randomly distributed across the genome

Utilizing the methodology outlined in Fig. 1B, we mapped endogenous and formaldehyde-induced DPCs in human fibrosarcoma HT1080 cells (DPC-seq). In this approach, protein-crosslinked DNA fragments are selectively precipitated from highly sheared chromatin in the presence of KCl and SDS (Supplementary Fig. S1). DPC-containing DNA fragments are then subjected to de-crosslinking, adaptor ligation, PCR amplification, and next-generation DNA sequencing (see the “Materials and methods” section and Supplementary Figs S2 and S3). The identified DNA sequences were mapped to the human genome to reveal DPC locations.

As an initial step, ChromHMM, a computational tool that correlates chromatin marks with functional elements and regulatory activities of the genome [34–36], was employed to examine the formation patterns of formaldehyde-induced DPCs. This 18-state model provides a comprehensive annotation of the genome, where each state has a unique combination of histone modification marks, such as tri-methylation of lysine 4 of histone H3 (H3K4me3, a post-translational modification associated with chromatin at transcriptionally active loci) and tri-methylation of lysine 27 of histone H3 (H3K27me3, associated with chromatin at transcriptionally repressed regions), as well as DNA accessibility. To address the question of whether DPCs are formed randomly across the genome, we obtained publicly available HT1080-specific ChromHMM segmentation (accession number ENCFF461DZD).

The first step was to analyze the distribution of DPC sites across the genome in untreated control samples to detect endogenous levels of DPCs. Peak lists for untreated control samples were identified using MACS2, then the fold-enrichment between each chromatin state and untreated control peak lists was computed for three independent replicates (see the “Materials and methods” section for details). The analysis shows an essentially uniform distribution across each of the chromatin states (Fig. 2A, three columns labeled ‘C’; Supplementary Table S2).

We subsequently examined samples treated with 400 μM formaldehyde for 2 h (T), a condition selected based on previously published data [25] and demonstrated a significant induction of chromosomal DPCs at this concentration (Supplementary Fig. S1), using the approach outlined earlier. We hypothesized that distribution of DPCs present immediately following formaldehyde treatment would exhibit a non-random distribution compared to untreated controls. In contrast to the untreated control samples, DPCs in treated samples were significantly enriched within specific subdomains of the genome. This non-random pattern of DPC formation was consistently observed across three independent replicates (Fig. 2A, three columns labeled T; Supplementary Table S2). The heatmap reveals that Polycomb-repressed genes, bivalent TSSs, and bivalent enhancers exhibit the highest signals. In addition, strong transcription, weak enhancer, and zinc-finger (ZNF) genes and repeats chromatin states showed a significant increase in DPC signal immediately post-formaldehyde exposure. A correlation analysis was performed between DPC-seq signals at the initial treatment and data from ATAC-seq, RNA polymerase II, and various histone marks of HT1080 cells. The analysis showed a positive correlation, indicating that formaldehyde-induced DPCs tend to preferentially form in accessible chromatin regions (Supplementary Fig. S4).

To visualize the differences in patterns of DPC formation between untreated (control; C) and formaldehyde-treated samples (T), normalized reads for the two treatments were plotted using Integrative Genomics Viewer [37] (Supplementary Fig. S5). An 83 Mb segment of chromosome 17 was selected due to its gene-rich nature. The control signal (depicted in black), representing endogenously formed DPCs, exhibited an essentially uniform distribution across this portion of the human genome, in all three replicates. In contrast, reads from formaldehyde-treated (T) cells (Supplementary Fig. S5, depicted in blue) displayed a distinct pattern compared to the untreated control samples. This analysis further supports the interpretation that formaldehyde treatment results in a non-random distribution of chromosomal DPCs.

### DPC removal is regioselective

We hypothesized that DPC removal would occur in a regio-selective manner. To test this prediction, an analysis similar to that outlined above was performed on HT1080 cells that had been exposed to formaldehyde for 2 h and permitted to recover in drug-free media for five additional hours, a timepoint that was chosen based on previously published data [25] (Supplementary Fig. S2). The data presented in Fig. 2A (three columns labeled R; Supplementary Table S2) reveal a significant reduction in DPC signal after the 5-h recovery period in active TSSs, transcriptional regions, and enhancer states. In contrast, DPCs persisted in transcriptionally inactive regions, such as heterochromatin, Polycomb-repressed genes, bivalent TSS, and bivalent enhancers. The visual representation of normalized reads using Integrative Genomics Viewer at an 83 Mb scale revealed no appreciable difference in the distribution of DPCs within these respective chromosomal domains when samples were recovered immediately post-formaldehyde treatment (T) or following a 5-h drug-free recovery (R) period (Supplementary Figs S5 and S6A in light blue). At a 500 kb scale, selective removal of DPCs is clearly occurring at loci undergoing active transcription as indicated by the ChromHMM track (Fig. 2B and Supplementary Fig. S6B).

To further confirm that DPCs formed in genes undergoing RNA polymerase II-mediated transcription are more efficiently removed than those formed at transcriptionally quiescent loci, we performed the following experiment. Publicly available polyA plus RNA-seq data of HT1080 cells [31] was accessed and the gene expression levels were divided into five quintiles (4630 genes per quintile). These quintiles were organized from Q0, composed of non-expressed genes, to Q4 composed of the most highly expressed genes, with Q1–Q3 being composed of genes with levels of transcriptional activity between these two extremes. The validity of these quintiles was confirmed by overlapping their TSSs with the signal intensity of histone H3 lysine 4 trimethylation (H3K4me3) signal, a histone modification that marks the TSS of active genes [38]. The H3K4me3 signal was plotted as a heatmap, with each quintile centered at the TSS with a 20 kb window upstream and downstream (Fig. 3A). Next, DPC-seq data generated from material recovered from cells at the T (immediately following a 2-h formaldehyde treatment) and R (5-h drug-free recovery) periods were plotted as normalized reads. The graphs on top of the heatmaps depict the signal intensity for each quintile. Immediately following formaldehyde treatment (T), the signal of DPC reads across all quintiles overlapped and showed a similar baseline (Fig. 3B, left heatmap).

In contrast, after a 5-h drug-free recovery (R), the signal became stratified based on transcriptional activity, with highly transcribed genes (Q4, green) showing lower DPC levels compared to poorly transcribed genes (Q1, red). These data show that the loss of DPC signal is most pronounced at the transcription start site and tracks with the direction of transcription (Fig. 3B, right heatmap). It is noteworthy that the signal of DPCs at non-expressed genes (Q0, gray) remained relatively unchanged during the drug-free recovery period. These data, which strongly establish accelerated DPC removal at actively transcribed genomic regions, are consistent with a similar analysis performed by Oka *et al.* [27].

### Reprogramming transcription alters DPC removal efficiency

The synthetic corticosteroid dexamethasone functions as a transcription factor that modulates gene expression by interacting with its receptor and selectively binding to gene promoter regions [39]. We hypothesized that altering transcriptional activity with dexamethasone would reprogram DPC removal dynamics. We chose to focus on IL1B, a gene that has been reported to be actively transcribed in dexamethasone-free conditions but becomes transcriptionally repressed following dexamethasone treatment in HT1080 cells [33]. As a control, we utilized RT-qPCR to confirm IL1B expression was dexamethasone dependent in our HT1080 cells (Supplementary Fig. S7).

We predicted that the efficiency of DPC removal from this gene would be substantially reduced following dexamethasone treatment. To test this hypothesis, HT1080 cells were pre-treated with 1 μM dexamethasone overnight, followed by a 2-h 400 μM formaldehyde treatment and a 5-h formaldehyde-free recovery period (during which time dexamethasone was continuously present). Subsequently, recovered DNA was sheared, and DPCs were isolated using KCl/SDS precipitation as described in the “Materials and methods” section. Deproteinized DNA was then subjected to a DPC-selective qPCR with IL1B gene-specific primers (Fig. 4A and Supplementary Table S1). The data depicted in Fig. 4B reveal a significant (*P =*.004) two-fold reduction in DPC removal from the IL1B locus following dexamethasone treatment, robustly demonstrating that transcriptional reprogramming indeed alters the efficiency of DPC removal. To control for the possibility that dexamethasone directly represses DPC repair, we performed an additional experiment examining DPC removal kinetics at a locus (RIPOR2) that becomes transcriptionally activated upon dexamethasone treatment [33]. We used the DPC-selective qPCR technique with RIPOR2-specific primers and observed a higher percentage of formaldehyde-induced DPC removal in dexamethasone-treated samples compared to untreated control (data not shown). These data suggest that dexamethasone treatment does not inhibit the repair of DPCs.

### Transcription-coupled DPC removal is impaired in NER-deficient cells

Since cells utilize transcription and replication as surveillance mechanisms to detect DNA damage and initiate damage recognition and response pathways [40, 41], we predicted that a transcription-coupled repair could be involved in the removal of DPCs. We and others have shown that cells deficient in NER have an impaired ability to repair DPC lesions present on transfected substrates [42–44]. Since NER has a sub-pathway that is transcription-coupled [45, 46], we suspected that this TC-NER pathway contributes to the removal of formaldehyde-induced chromosomal DPCs. To test this hypothesis, we isolated NER-deficient HT1080 cells from XPA gene knockout pools generated using CRISPR technology (XPA-KO, see the “Materials and methods” section, and Supplementary Figs S8 and S9) that have mutations in the XPA gene. This gene encodes the xeroderma pigmentosum protein A, a critical damage-verification and scaffolding repair factor in the NER pathway [47, 48]. We predicted that, due to the loss of transcription-coupled NER, XPA-KO cells would exhibit diminished removal of DPCs from highly transcribed genes, compared to their unmodified WT counterparts.

To test this hypothesis, DPC-seq was performed with WT and XPA-KO cells. Heatmaps of the DPC-seq immediately following a 2-h formaldehyde treatment (T) and after a 5-h drug-free recovery period (R), merged from two independent replicates, were generated as described earlier. We examined the distribution of DPCs across the Q0–Q4 gene sets (4195 genes per quintile) described earlier. Analysis of the DPC signals from samples harvested from cells immediately following formaldehyde treatment across the transcription start sites of these quintiles revealed no apparent difference when WT and XPA-KO cells were compared (Supplementary Fig. S10). We next compared patterns of DPC distribution in formaldehyde-treated WT and XPA-KO cells following a 5-h recovery period. Consistent with results obtained in the series of experiments described in Fig. 3, we observed that, in WT cells, DPCs were removed with higher efficiency from actively transcribed genes as compared to poorly or non-transcribed genes (Fig. 5A, left heatmap). Interestingly, while XPA-KO cells displayed evidence of transcription-coupled removal of DPCs, the extent of DPC removal from transcribed loci appeared smaller than that observed in WT cells (Fig. 5A, right heatmap). To test if this difference in DPC removal between the WT and XPA-KO cells was statistically significant, we calculated the average signal intensity at Q0, as an internal control, and Q4 quintiles for replicates 1 and 2 (Supplementary Fig. S11). The data show that the Q0 signals of the two cell lines are indistinguishable, indicating that DPC removal from non-transcribed loci occurs at similar efficiencies in NER-proficient and NER-deficient cells. In contrast, the Q4 signal is substantially lower in the WT compared to the XPA-KO cells (Fig. 5B), indicating that the former cells remove DPCs from transcribed loci more efficiently than do the latter. The log-scaled *P*-value plot depicted in Fig. 5C confirms that while there is no significant difference in the efficiency with which the WT and XPA-KO cells remove DPCs from non-transcribed loci (gray line), there is a significant difference in the efficiency with which WT and XPA-KO cells remove DPCs from highly transcribed loci (Fig. 5C, green line). These results are consistent with the interpretation that transcription-coupled removal of formaldehyde-induced DPCs is diminished in NER-deficient cells.

We performed an additional series of experiments to confirm the prediction that DPC removal from a highly transcribed locus would occur less efficiently in XPA-KO cells, compared to WT HT1080 cells. Using the DPC-selective qPCR method schematically depicted in Fig. 4A, we found (Fig. 6, left panel) that formaldehyde-induced DPCs were removed from the IL1B locus significantly less efficiently in XPA-KO cells compared to WT cells (40% and 72%, respectively, *P*= .002). Complementing XPA-KO cells with XPA gene restored the efficiency of DPC removal in these cell lines (Fig. 7 and Supplementary Fig. S9A).

To further confirm the defect in DPC removal from this locus in NER-deficient genetic background, we performed an additional series of experiments in which the WT and XPA-KO cells were treated with the nitrogen mustard drug mechlorethamine, another agent known to induce chromosomal DPCs [49] (Supplementary Fig. S12). These experiments showed that XPA-KO cells were deficient in the removal of mechlorethamine-induced DPCs from the IL1B locus as compared to WT cells (21% and 66% removal, respectively, *P*= .001) (Fig. 6, right panel). Taken together, the data in Figs 5 and 6 support the interpretation that transcription-coupled NER plays a role in the removal of xenobiotic-induced chromosomal DPCs. This interpretation is consistent with previously published findings from our group and others, showing that DPCs present on transfected DNA were more efficiently repaired when the locus containing the lesion was actively transcribed [43, 44].

To confirm the involvement of TC-NER in DPC removal, we next inactivated the CSB (ERCC6) gene in HT1080 cells—an ATP-dependent DNA translocase that interacts with stalled RNA polymerase II—and examined the relative ability of these CSB-KO cells to remove formaldehyde-induced DPCs, compared to WT and XPA-KO cells. As expected, based on recent findings [27–29], we observed a significant reduction of DPC removal from the IL1B locus in CSB-KO cells, compared to WT cells (Fig. 7). It is noteworthy that while both of the knockout clones were significantly less able to remove formaldehyde-induced DPC, the magnitude of the defect observed in CSB-KO clones was substantially greater than that observed in the XPA-KO cells.

XPA-KO cells are deficient in both TC-NER and global genome (GG)-NER. To specifically assess the role of TC-NER in DPC removal, we utilized XPC-KO HT1080 cells, which are defective in the GG-NER but proficient in the TC-NER. Using the DCP-selective qPCR targeting the highly transcribed gene IL1B, we observed no significant difference in DPC removal between WT and XPC-KO cells (Fig. 7). This finding indicates that DPC removal at this transcriptionally active locus depends on TC-NER, not GG-NER.

### DPC removal from ribosomal DNA

Genomic DNA encoding ribosomal RNA (rDNA) is located on the short arms of the acrocentric chromosomes 13, 14, 15, 21, and 22 and as a separate 5S gene cluster present on chromosome 1. Each of these loci contains multiple copies of rDNA arranged in tandem arrays. Due to the repetitive nature and high copy number of rDNA genes, they are poorly annotated, and consequently examination of DNA repair activity at these loci cannot easily be accomplished via next-generation sequencing-based methodologies such as those we describe earlier. However, Yang *et al.* [32] used the canonical sequence of 45S pre-ribosomal N5 to devise a method that permitted them to map repair events generated from XR-seq of UV and cisplatin lesions to the rDNA, and provided compelling evidence that repair of UV- and cisplatin-induced lesions in rDNA was subject to global-genome NER, but not transcription-coupled NER [32].

We pursued a similar approach and re-analyzed the thus-filtered DPC-seq reads obtained from the experiment depicted in Fig. 2B. Determination of the number of reads mapping to the rDNA genes, as well as those mapping to the Q0 (non-transcribed) and Q4 (very actively transcribed) gene sets, was performed on material obtained from cells immediately following a 2-h treatment with 400 μM formaldehyde (T samples) as well as on material obtained from cells similarly treated with formaldehyde that had been permitted to recover for 5 h in drug-free media (R samples). Dividing the average number of mapped reads in the T samples by the number of mapped reads in the R samples yields the RRE value. The RRE quantitatively represents the efficiency with which DPC removal occurs at any selected locus (or loci) compared to the genome average. Thus, an RRE of >1, indicates that DPC removal occurs preferentially at this location, whereas an RRE of ∼1 indicates there is no preferential DPC removal at that locus (see the “Materials and methods” section for additional details). Based on the results from Yang *et al.* [32], we predicted that DPCs present within rDNA would not be subject to transcription-coupled NER, and consequently, the RRE for rDNA would closely resemble that for the Q0 gene set, i.e. be at or close to 1. We further anticipated seeing efficient removal of drug-induced DPCs from the Q4 genes. The results presented in Fig. 8A confirm these predictions. DPC removal efficiency at both Q0 and Q4 loci mirrors the analysis shown in Fig. 3; DPC removal was more efficient from loci in the highly transcribed (Q4) gene set than in poorly transcribed (Q0) gene set (Fig. 8A). Interestingly, DPC removal from the rDNA loci was relatively inefficient and roughly on par with that observed at the Q0 loci (Fig. 8A). A similar analysis was done in which 50 bp single-end reads were mapped to a reconstructed genome that only includes the canonical rDNA sequence, and 100 Q0 and Q4 control genes, and the same result was obtained (Supplementary Fig. S13). Our analysis shows that while DPCs in highly transcribed genes are efficiently removed, the removal efficiency at the rDNA loci is more consistent with that seen at non-transcribed genes. Our observation is consistent with the previous report indicating that UV and cisplatin lesions at the rDNA are not subject to transcription-coupled repair [32].

We reasoned that it would be possible to further test the hypothesis that NER does not significantly contribute to removal of DPCs from rDNA loci by performing a modified version of the DPC-selective qPCR technique that was employed above to examine DPC removal dynamics from the IL1B locus (Figs 4 and 6). In order to sample across the entirety of the rDNA loci, we modified this technique by using three sets of previously validated rDNA primers, each selective, respectively, for the 18S, 28S, or 5S genes (Supplementary Table S1) to amplify protein-linked rDNA [50]. DPC-containing DNA was recovered from WT and XPA-KO HT1080 cells immediately after formaldehyde treatment (T) as well as following a 5-h drug-free recovery period (R). Results from this analysis show that there was no significant difference in the efficiency of removal of DPCs from rDNA loci between WT and XPA-KO cells (Fig. 8B).

## Discussion

We used genome-wide next-generation sequencing combined with qPCR-based targeted analysis of specific chromosomal loci to examine the dynamics of spontaneous and formaldehyde-induced DPCs in WT and NER-deficient human HT1080 cells. This effort contributes to a growing body of literature in recent years that utilized unbiased next-generation sequencing-based approaches to examine the chromosomal dynamics of a variety of DNA lesions, including UV-induced photoproducts, cisplatin DNA–DNA crosslinks, and thymidine glycol adducts [17–22]. Our analysis supports the following conclusions: (i) steady-state levels of chromosomal DPCs are essentially evenly distributed across the genome, (ii) formaldehyde-induced DPCs form preferentially at sites associated with transcription and at more accessible chromatin regions, (iii) DPC removal occurs preferentially from RNA polymerase II-transcribed loci and correlates with transcriptional activity levels, (iv) formaldehyde-induced DPCs are repaired via transcription-coupled NER, and (v) DPCs induced within rDNA are not subject to transcription-coupled repair.

The observed non-random distribution of formaldehyde-induced DPCs at transcriptionally active sites and accessible chromatin regions (Fig. 2A and Supplementary Fig. S4) is possibly due to increased solvent accessibility and the proximity of cellular proteins to the DNA; however, the biological significance of this distribution remains unclear. Previous studies reported that actively transcribed sites are more susceptible to the formation of DNA single-strand breaks induced via ionizing radiation [51, 52], which aligns with the observed pattern of formaldehyde-induced DPC formation. Unlike UV-induced DNA photoproducts, which preferentially form at pyrimidine-rich regions [17], formaldehyde-induced DPCs do not seem to follow a sequence-specific pattern; rather, it is affected by the chromatin structure.

**Figure 9.**
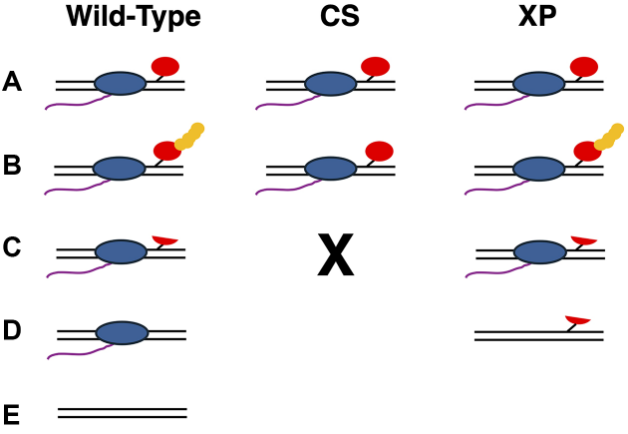
Model of transcription-coupled DPC repair. Key: parallel black lines, DNA duplex; blue oval, RNA polymerase II; red oval and stick, DNA-crosslinked protein; yellow balls, polyubiquitin chain conjugated onto DPC; purple line, mRNA transcript. See text for details.

While this work was in the final stages of completion, a series of papers were published that explored the dynamics of xenobiotic-induced DPCs in human cells. Broadly speaking, the results presented herein are consistent with these investigators’ major finding, which was that in human cells the Cockayne syndrome (CS) group A and B proteins are required to initiate transcription-coupled DPC repair. However, due to differences in experimental design, our results lead us to reconsider these investigators’ other major interpretation that NER factors that act downstream of the CS proteins, i.e. those required for transcription-coupled repair of UV photoproducts, do not contribute to this transcription-coupled DPC repair pathway [28, 29]. Below, we present a model (Fig. 9) that synthesizes these recently published observations with the findings described herein. The key element of this model is that transcription-coupled DPC repair in mammalian cells occurs *via* two distinct steps. First, DNA-crosslinked proteins encountered by the RNA polymerase II complex become polyubiquitinated and are subsequently enzymatically processed by the proteasome to produce DNA–peptide crosslinks. Second, the remaining DNA–peptide product is excised via the TC-NER machinery.

A role for the CSB gene in DPC repair was first described by Burgos-Moron *et al.* in 2018 [53], and more recently a trio of papers described in significant detail the role of this protein in transcription-coupled repair of formaldehyde-induced DPCs [27–29]. Consequently, as illustrated in Fig. 9, left panel, our model proposes that transcription-coupled DPC repair is triggered by RNA polymerase II stalling at the site of the lesion (A, Fig. 9). This stalling activates a CSB gene product-dependent signal transduction cascade resulting in poly-ubiquitination of the DNA-crosslinked protein (B, Fig. 9). In WT cells, polyubiquitination of the DNA-crosslinked protein triggers a proteasome-mediated proteolysis of the DNA-crosslinked protein, leading to the production of a remnant DNA–peptide crosslink, depicted as a small red arc attached to the DNA backbone (C, Fig. 9). Finally, the peptide fragment crosslinked to DNA is excised via the NER machinery, resulting in the final step of DPC repair (D, Fig. 9), leading to a resumption of RNA polymerase II transcription, leaving behind a fully repaired DNA duplex (E, Fig. 9). In contrast, in CSA- or CSB-deficient clones (“CS,” middle panel, Fig. 9), this signal transduction pathway is not activated, the crosslinked protein doesn’t undergo polyubiquitination, or proteasome-mediated degradation, and consequently RNA polymerase II remains stalled at the site of the DPC, ultimately leading to elevated levels of cell death (“X,” Fig. 9C). In cells deficient in the TC-NER factors that act downstream of the CS proteins (e.g. XPA, “XP,” right panel, Fig. 9), our model proposes that while polyubiquitination and proteasome processing occur, the subsequent DNA–peptide crosslink cannot be excised. Importantly, we propose that the remaining DNA–peptide lesions are subject to RNA polymerase II bypass transcription (D, Fig. 9). This model is critically dependent on the data presented herein. Importantly, however, it is also supported by a substantial body of literature on mammalian DPC repair (summarized below). In addition, the model contains features that reconcile several seemingly paradoxical findings. These are discussed in some detail below.

First, the model incorporates recent findings [27–29] that CSA and CSB clones are deficient in transcription-coupled DPC repair. These findings extended an earlier report [53] that concluded that the Cockayne syndrome group B protein was required to repair transcription blocks induced by the DPC-forming agent 5-aza-2′-deoxycytidine. The model integrates the findings of several studies showing proteasome-mediated processing of DPCs [27–29, 42, 44, 54–57]. Second, the model is consistent with numerous publications showing that DPCs present on substrates transfected into mammalian cells, or that form on chromosomal DNA, are repaired via an NER-dependent mechanism [42–44]. Third, the model provides an attractive resolution to the apparent paradox that while repair of DPCs comprised of large proteins occurs through an NER-dependent process [42–44], it has been repeatedly confirmed that the NER machinery, while capable of excising DNA peptide crosslinks cannot process larger DPCs [44, 58–60]. As our model proposes, the most likely explanation is that during transcription-coupled DPC repair, the proteasome (activated by CSA/CSB-dependent polyubiquitination of the crosslinked protein) processes DNA-crosslinked proteins to substantially smaller products that are susceptible to NER machinery-mediated removal. The length of the initial crosslinked peptides that remain covalently attached to chromosomal DNA is yet to be determined, however, studies indicate that free, i.e. non-DNA-crosslinked proteins that have been subject to proteasomal degradation are generally reduced to a collection of 3–30 amino acid peptides [61]. The human 26 S and 20 S proteasomes generate overlapping but different sets of peptide fragments from a model protein substrate [62]. It therefore seems likely that the remaining DNA peptide crosslinks are likely to be between several hundred and several thousand Daltons in molecular weight. As noted earlier, while specific findings varied, *in vitro* excision studies have repeatedly confirmed that the NER machinery is capable of excising crosslinked peptides in this size range [44, 58, 60]. The heterogeneity in the size of the remaining DNA-crosslinked peptides provides a plausible explanation for why we observed a greater defect in removal of formaldehyde-induced DPCs in CSB-KO cells compared to XPA-KO cells (Fig. 7). We propose that only a subset of the proteasome-processed DPCs is sufficiently long to be precipitated by KCl/SDS treatment, and thus only a fraction of the unrepaired DNA peptide crosslinks that accumulate in XPA-KO *cells* will be captured by the DPC-selective qPCR assay. In contrast, it seems likely that essentially all the full-length DPCs that accumulate in CSB-KO cells will be subject to KCl/SDS precipitation.

Intriguingly, Nakano *et al.* [58] reported that while XPA-deficient clones displayed no defect in the removal of formaldehyde-induced DPCs that were greater than ∼8 kDa, these cells were essentially unable to remove smaller DNA–peptide crosslinks—lesions that were rapidly removed from WT cells. While it is conceivable that these latter molecules are created when low-molecular-weight nuclear peptides become crosslinked to chromosomal DNA, we believe it much more likely that these DNA–peptide crosslinks accumulate in XPA-deficient cells, due to the excision repair defect in the XPA cells. Fourth, this model provides a compelling explanation for the differential phenotype of cells deficient in the CS proteins versus cells deficient in NER proteins known to be required for excision of UV-induced photoproducts.

In addition, our model provides a compelling alternative explanation for observations that led others to rule out a role for the NER machinery in chromosomal transcription-coupled DPC repair. The first of these findings was that CSA- and CSB-deficient cells are hypersensitive to death induced by the DPC-forming agent 5-aza-2′-deoxycytidine [28, 29, 53], while numerous clones deficient in “canonical NER,” including XPA, XPC, XPF, and XPG, are not [28, 29], and the second was that CSA- and CSB-deficient cells display a prolonged blockade of RNA polymerase II transcription following acute exposure to formaldehyde that is not observed in NER-deficient clones [27–29]. As our model outlines, we propose that the explanation for both these apparent paradoxes lies in the different biological consequences associated with the accumulation of large cellular proteins (such as occurs in CSA/CSB-deficient cells following treatment with a DPC-inducing agent), compared to the accumulation of far smaller DNA–peptide crosslinks (such as our model proposes occurs in XP-gene deficient cells following similar drug exposure). Our model proposes that the signalling cascade that leads to polyubiquitination and proteasome degradation of DPCs encountered during transcription occurs in both WT and XP cells but does not occur in CSA/CSB-deficient cells. The accumulation of these full-length DNA-crosslinked proteins in these latter cells, blocks RNA polymerase II transcription, and leads to elevated levels of programmed cell death, thereby explaining the hypersensitivity of these clones to 5-aza-2′-deoxycytidine-induced cell death.

In contrast, we propose that in XP-deficient cells, the initial phase of transcription-coupled DPC repair is functional, resulting in conversion of the DNA–protein crosslink to much smaller DNA–peptide crosslinks. Based on previous work done by us [63], we predict that these lesions do not block RNA II polymerase and bypass transcription, and thus, do not trigger programmed cell death, thereby explaining why the XP-deficient clones are not hypersensitive to cell death induced by 5-aza-2′-deoxycytidine. This prediction is consistent with findings that while DNA-crosslinked histone H2A (14 kDa) or alkylguanine DNA alkyl transferase (AGT, 53.1 kDa) completely blocked T7 RNA polymerase transcription, smaller DNA–peptide crosslinks containing between 10 and 57 amino acids (∼1.2–6.7 kDa) only very modestly reduced transcription efficiency [64]. Ji *et al.* [64] also found that transcription through these DNA–peptide crosslinks occurred with remarkably high fidelity, although they did note that the precise chemical nature of the DNA–peptide crosslink exerted an effect on transcription fidelity. A similar study performed by a different group observed a more potent inhibitory effect of DPCs on T7 fidelity RNA polymerase transcription efficiency; however, this group also observed that the size of the crosslinked protein/polypeptide significantly influenced the magnitude of this effect [65]. While it remains to be experimentally confirmed, it is reasonable to propose that DPCs represent a far more significant barrier to RNA polymerase II transcription bypass than DNA–peptide crosslinks. However, such a conclusion would appear to be the most plausible explanation for why CSA/CSB-deficient clones exhibit a powerful formaldehyde-induced blockade of RNA polymerase II transcription while XPA clones—which our data convincingly demonstrate accumulate KCl/SDS precipitable DNA lesions—do not.

While evoking a role for NER in the final step of repair provides a compelling and elegant explanation for how cells ultimately repair the products of the recently described CSB-dependent DPC processing pathway [27–29], several questions remain. It is unclear whether additional cellular DNA repair pathways, including recombinational repair can recognize proteasome-generated DNA–peptide crosslink products, presumably when they are encountered during DNA replication. Experiments are currently underway to identify the chemical structures of DNA-crosslinked protein and peptide residues that accumulate in XPA-KO cells that have been treated with formaldehyde, and to examine a potential role for homologous recombination in processing them. It will also be of interest to determine the extent to which recombinational repair contributes to the removal of unprocessed DPCs in CSB cells. Finally, Nakano *et al.* [58] presented evidence that DPCs comprised of small cellular proteins ∼10 kDa and smaller are likely to be directly acted upon by the cellular NER machinery. It will therefore be of interest to investigate this question further and to determine the extent to which it contributes to transcription-coupled (and transcription-independent) DPC repair in mammalian cells.

## Supplementary Material

gkaf720_Supplemental_File

## Data Availability

The data underlying this article are available in GEO and can be accessed with accession number GSE274678. To review GEO accession GSE274678, go to https://www.ncbi.nlm.nih.gov/geo/query/acc.cgi?acc=GSE274678. Data are also available to be viewed in UCSC browser with the following URL: https://genome.ucsc.edu/s/alsha088/DPCseq_NAR.

## Supplementary data

Supplementary data is available at NAR online.
