# Supplementary material for: Genome-wide mapping of formaldehyde-induced DNA–protein crosslinks reveals unique patterns of formation and transcription-coupled removal in mammalian cells
Source: Nucleic Acids Res. 2025 Jul 31;53(14):gkaf720. doi: 10.1093/nar/gkaf720 (PMC12311792; doi:10.1093/nar/gkaf720)
Supplement: gkaf720_Supplemental_File [file gkaf720_supplemental_file.docx]

**­­­Supplementary Figures**

**Figure S1. Selective enrichment of formaldehyde-induced DPCs with the ARK assay.** A) DPCs were selectively enriched using the ARK assay, followed by proteinase k digestion of the DNA-linked protein. Purified protein-linked DNA was run on 1% agarose gel for untreated control and a 2-hour 400 µM formaldehyde-treated samples in three independent replicates. B) Quantification of the gel in (A) measure as fold induction of DPCs. p=0.04, N= 3. Error bars represent SEM.


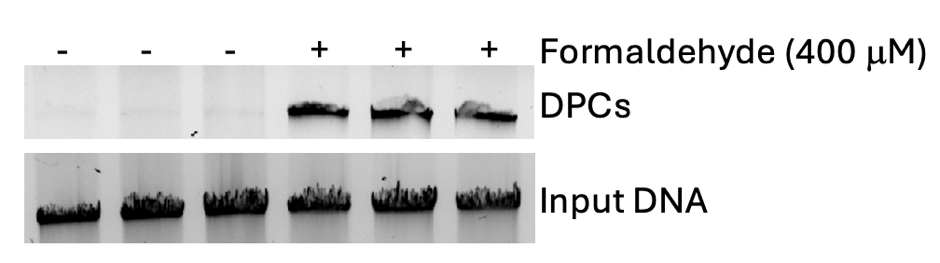

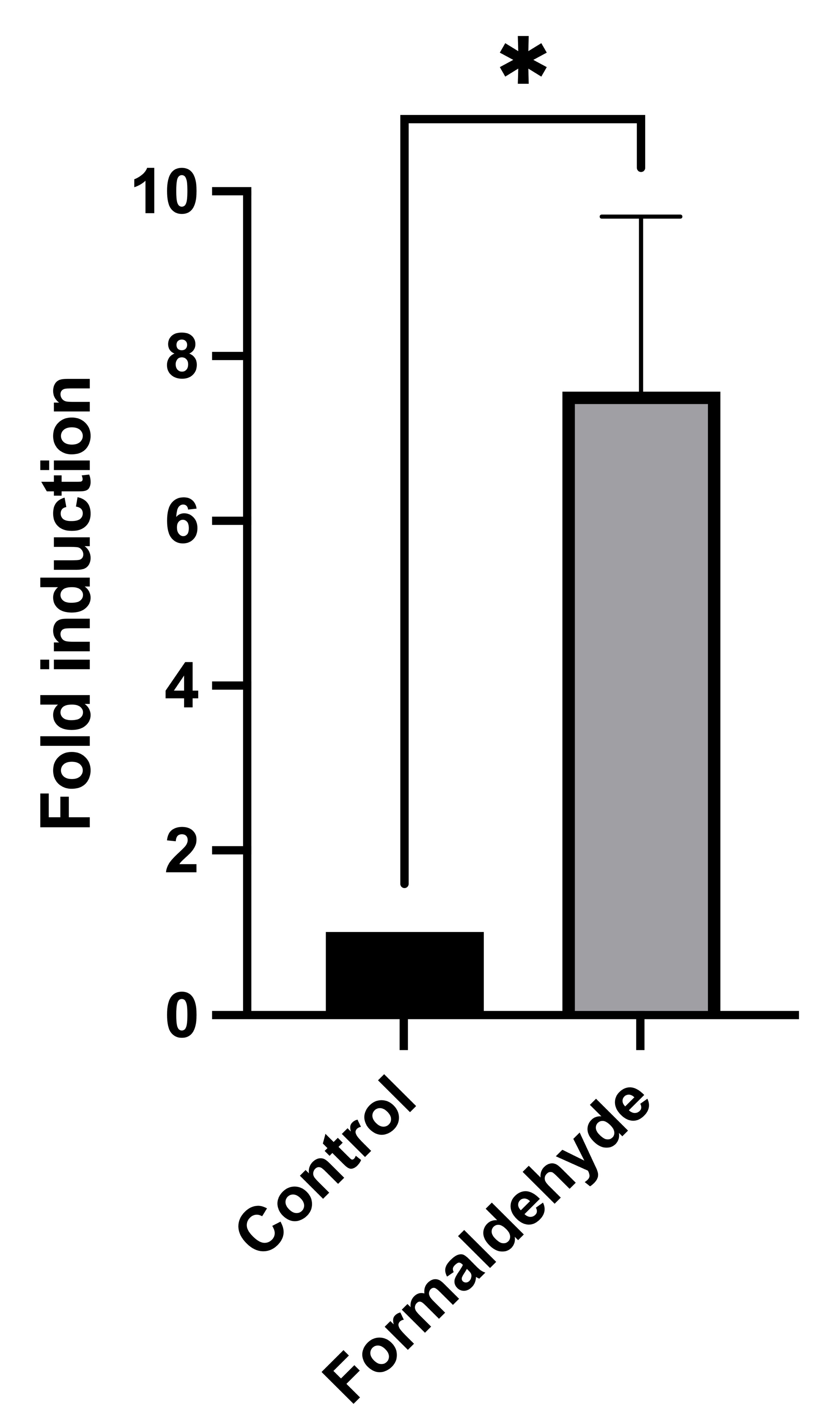


A)

B)

**Figure S2. Amount of precipitated protein-linked DNA.** Covaris sheared materials were subject to KCl-SDS precipitation, then amount of precipitated protein-linked DNA was measured with Qubit after being normalized to input for untreated control samples, as well as for samples collected immediately following a two-hour 400 µM formaldehyde treatment (T) and five-hour drug-free recovery (R). * p<0.02, N= 4. Error bars represent SEM.


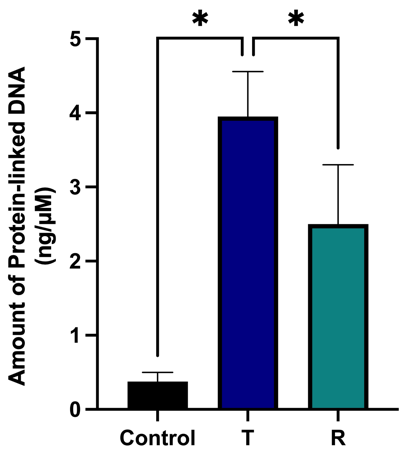


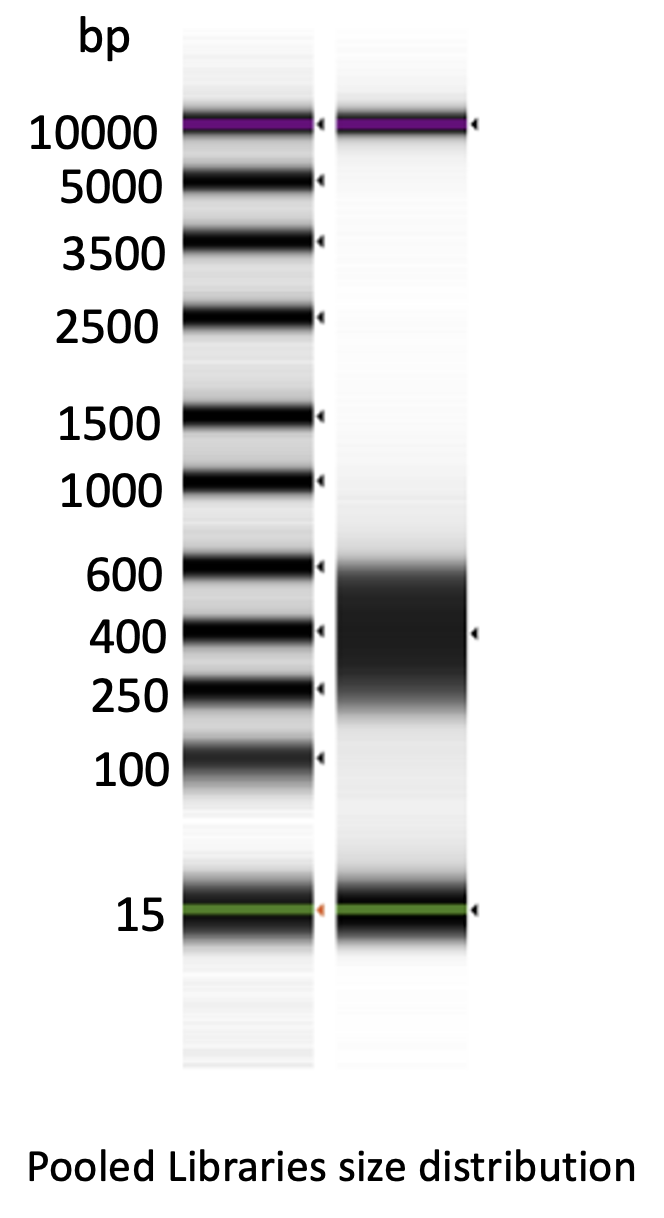


**Figure S3. Pooled library size distribution.** TapeStation electrophoresis as a QC for prepared DNA library. Lane 1, DNA marker; lane 2, size distribution of pooled libraries from untreated control, initial treatment and five-hour recovery samples.


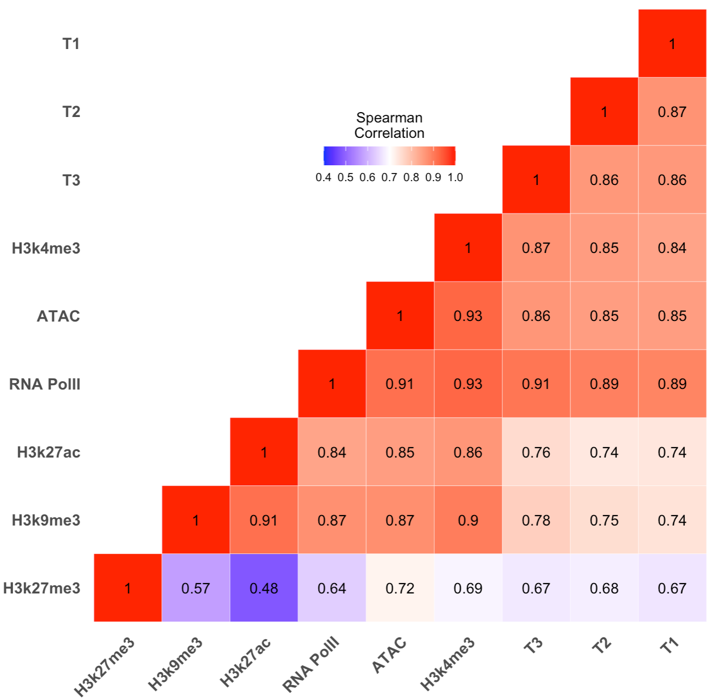


**Figure S4. Formaldehyde-induced DPCs are formed in accessible regions.** Spearman's correlation coefficient ρ calculated between DPC seq signal at initial formaldehyde treatment (T; three independently) and between ATAC seq, RNA polymerase II, and histone marks for transcription start sites (H3K4me3), enhancers (H3K27ac), heterochromatin (H3K9me3), and Polycomb repressed genes (H3K27me3). Darker red shades indicate higher correlation. While the Polycomb signal (H3K27me3) shows the lowest correlation, it remains relatively high. It worth noting that the correlation scale is tightly ranged from 0.4 to 1, with Polycomb showing a correlation of about 0.7 with the initial treatment peaks (T1-T3), compared to about 0.5 with enhancers (H3K27ac).

**Figure S5. Genome-wide patterns of formaldehyde-induced DPC formation and removal.** A) DPC seq tracks for untreated (C), two-hour formaldehyde treated (T), and five-hour recovery (R) samples at chromosome 17 with 83 mb scale. The y-axis represents normalized coverage, with group auto-scaling applied to all tracks (maximum value set to 0.25).


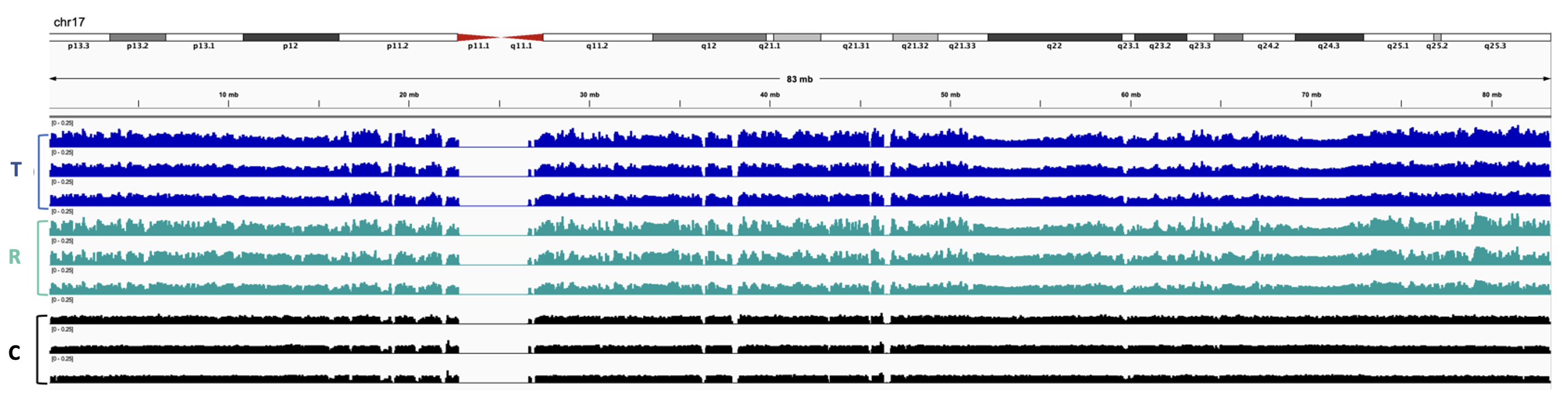


[This Photo](https://commons.wikimedia.org/wiki/File:Curved_Arrow.svg) by Unknown Author is licensed under [CC BY-SA](https://creativecommons.org/licenses/by-sa/3.0/)

**Figure S6. Patterns of formaldehyde-induced DPC formation and removal.** A) DPC seq tracks for untreated (C), two-hour formaldehyde treated (T), and five-hour recovery (R) samples at chromosome 3 with 18 mb scale. B) Zoom-into a fragment of chromosome 3 showing a 1000kb section demonstrates the selective removal of DPCs from transcribed genes as mapped to ChromHMM track in green. The green-colored regions in ChromHMM track indicate actively transcribed genes.


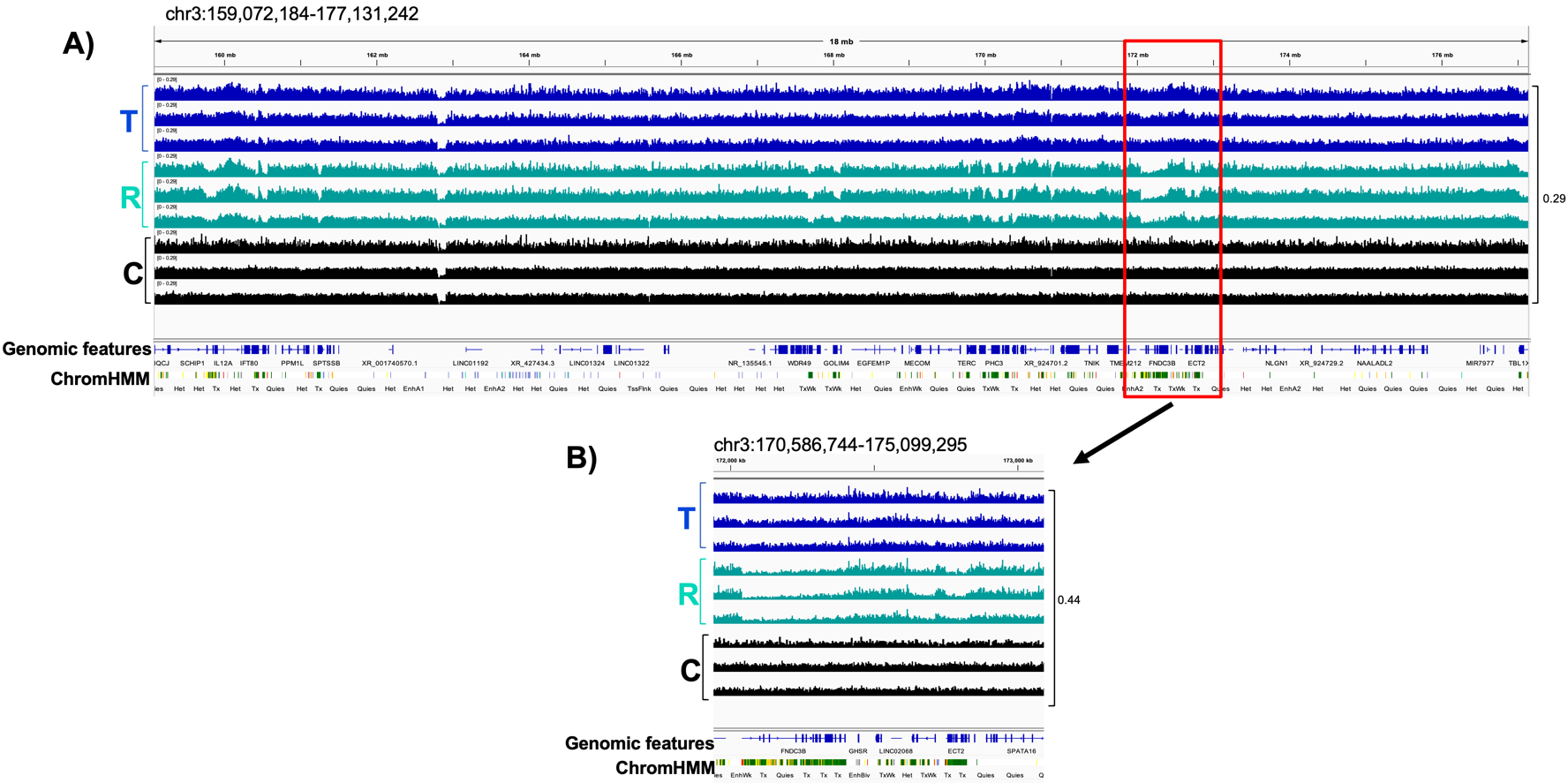


**Figure S7. Fold change of IL1B gene expression in the presence and absence of dexamethasone.** RT-qPCR was performed on WT HT1080 cells treated overnight with 1 μM dexamethasone. Dexamethasone treatment resulted in a 30-fold reduction of IL1B expression. Gene expression levels were normalized to the housekeeping gene GAPDH, and fold change was presented on a logarithmic y-axis. Fold change values: -Dex = 1, +Dex= 0.033.


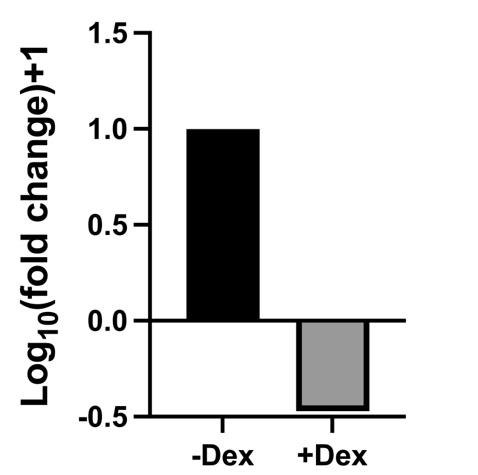


**Figure S8. Genotyping of WT and XPA-KO cells with XPA primers.** A) Agarose gel showing the PCR amplified XPA amplicon. The WT amplicon is 484bp and single-cell isolated XPA-KO colony shows a single band that is shorter than the WT one. B) Sanger sequencing of WT and XPA-KO amplicons from (A). The map shows the DNA sequence for WT and XPA-KO amplicons, the amino acids sequence for WT, and the locations of the three gRNAs used in the CRISPR KO. The sequences of the gRNAs are as follows: gRNA1: AGAGAUGGCGGCGGCCGACG; gRNA2: CGCAGCCGCCGUCGCCGAGU; gRNA3: CGGUGCGGGCGAGUAUCGAG


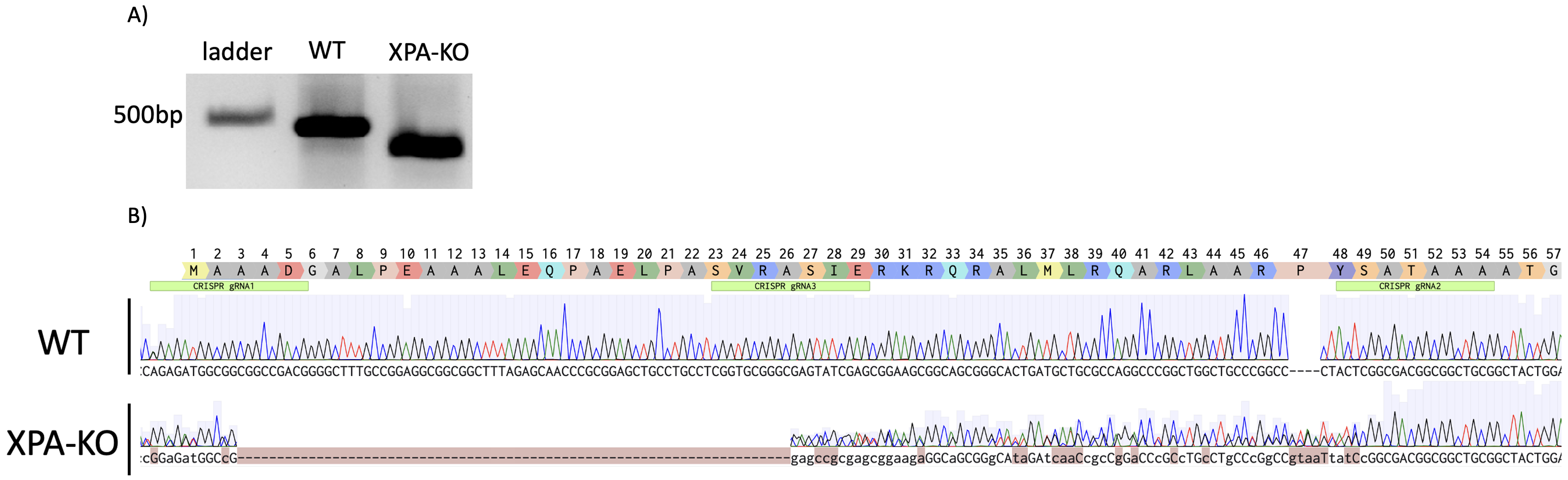


**Figure S9. Western blots for WT and KO cell lines.** Western blotting using antibodies against XPA (A), XPC (B), or CSB (C) for WT, XPA-KO, XPA-KO-corrected, XPC-KO, and CSB-KO HT1080 cells.


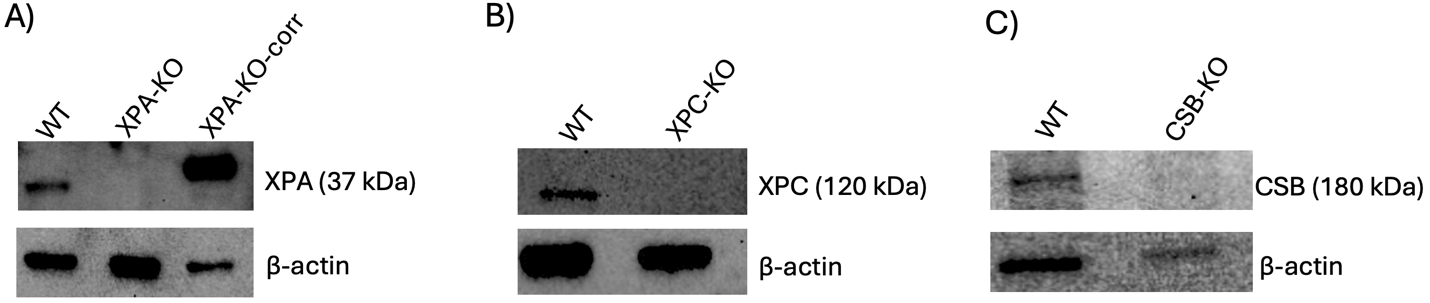


**Figure S10. DPC signal at initial treatment in WT and XPA-KO cells.** Heatmaps represent the signal intensity of H3K4me3 and DPC seq data merged from two independent replicates after a two-hour treatment with 400 µM formaldehyde (T) in WT and XPA-KO HT1080 cells across five quintiles of genes. Quintile Q0 represents non-expressed genes, while transcriptional activity increases from Q1 to Q4 as shown with the H3K4me3 signal. The quintiles are centered at the transcription start site (TSS) with a 20 kb window. The box above the heatmap depicts the signal intensity for each of the five gene sets.


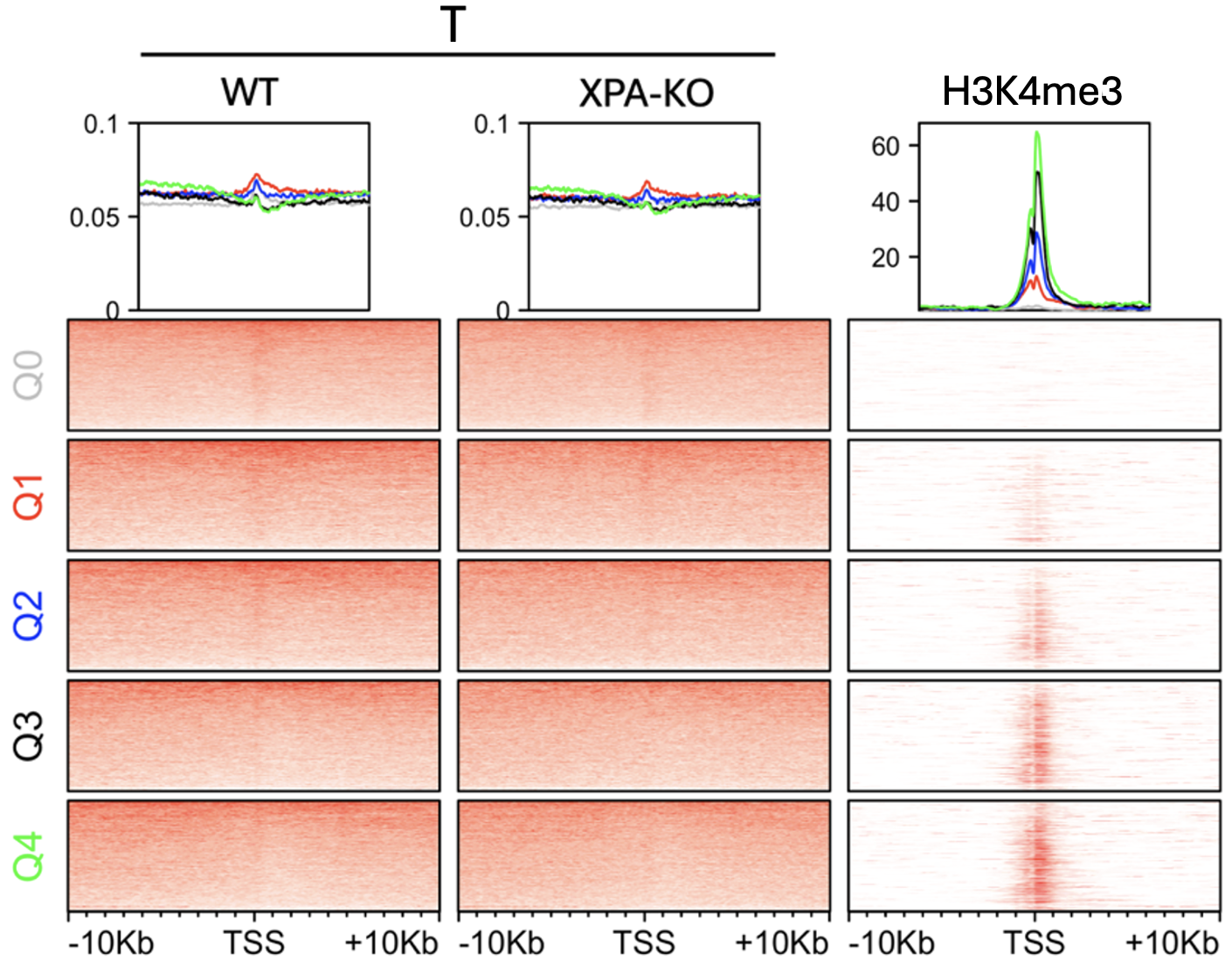


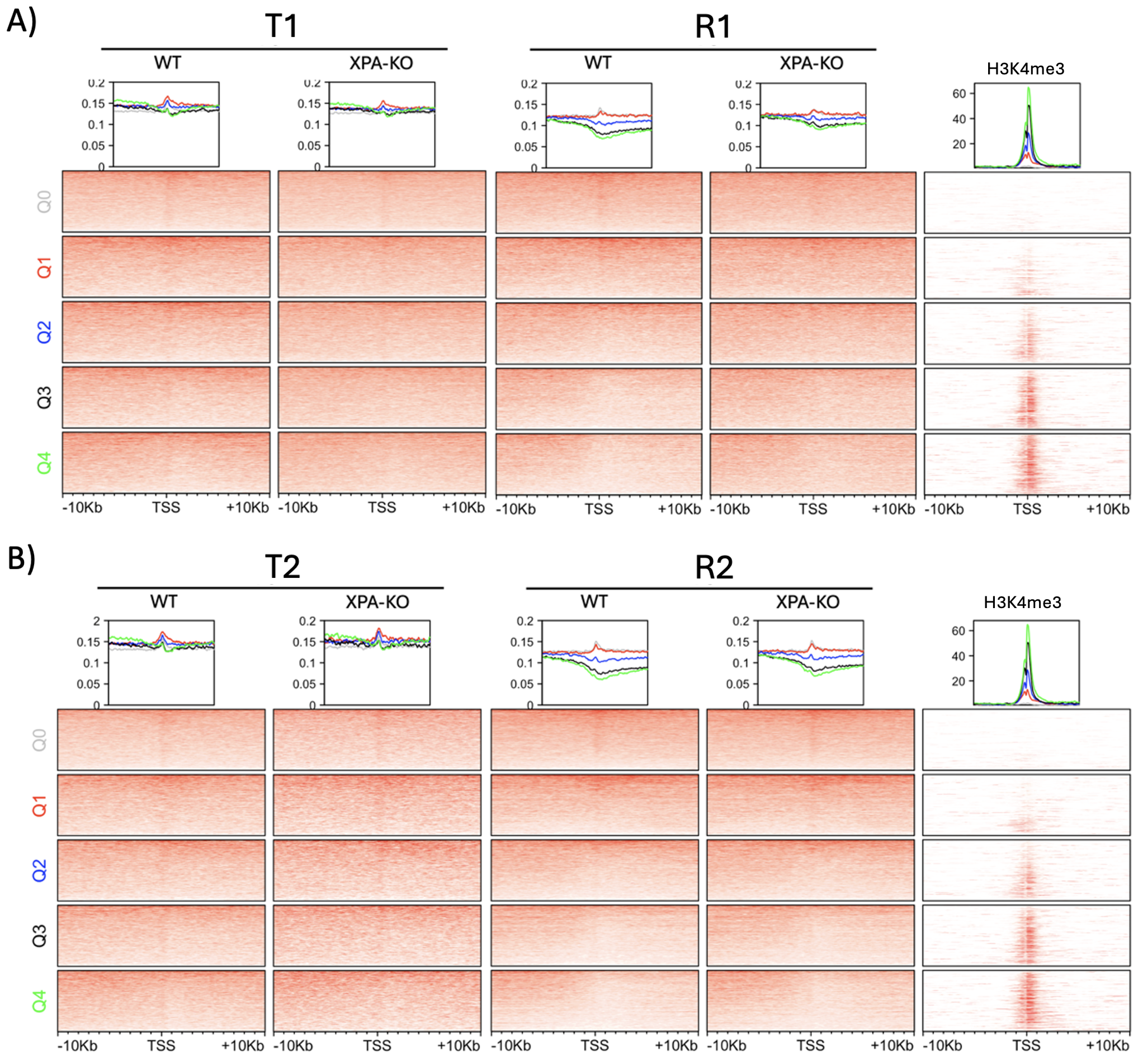


**Figure S11. DPC signal at initial treatment and after 5h recovery in WT and XPA-KO cells from each replicate.** Heatmap represents the signal intensity of H3K4me3 signal and DPC seq data from two independent replicates, replicate 1 (A) and replicate 2 (B), after a two-hour treatment with 400 µM formaldehyde (T), and a five-hour recovery period (R) across five quintiles of genes as explained in Fig. S10.

**Figure S12. Concentration-dependent formation of mechlorethamine-induced DPCs.** HT1080 cells were treated with increasing concentration of mechlorethamine then the ARK assay was performed to enrich for DPCs. Protein-linked DNA was subject to proteinase K digestion followed by agarose gel electrophoresis.


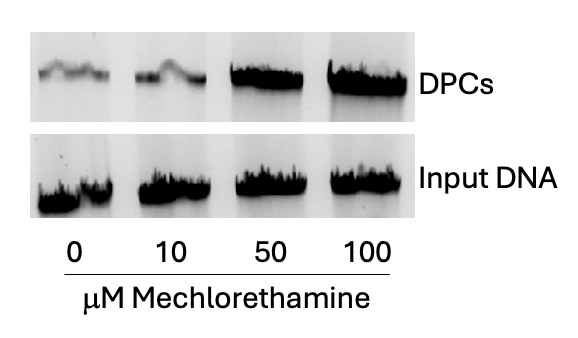


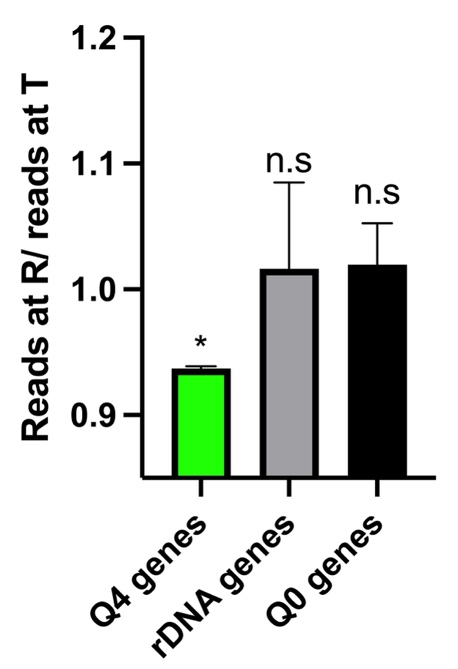


**Figure S13. rDNA analysis.** DPC reads of 50bp length was mapped to the reconstructed rDNA with 100 positive and negative control genes from Q4 and Q0; respectively. After filtering mismatches, fraction of reads at five-hour recovery samples was calculated as (reads at R/reads at T) and plotted. t test was done to compare number of reads at initial treatment (T) and five-hour recovery (R) in each gene set. **P =* 0.03; n.s.; not significant, N=3. Error bars represent SEM.

**Table S1. List of primers used in this study**

| **Name** | **Forward primer** | **Reverse primer** |
| --- | --- | --- |
| **Kapa primers** | AATGATACGGCGACCACCGA | CAAGCAGAAGACGGCATACGA |
| **IL1B** | AAACAAGAGTGCTGGAGCGA | AATGATGGGTTCACCGCACT |
| **XPA sequencing** | CGGGGAGAATCTGCACACAT | ATACCCAGACTCCGCCCAG |
| **IL1B-cDNA** | ACTGAAAGCTCTCCACCTCC | CTCCAGCTGTAGAGTGGGCT |
| **GAPDH-cDNA** | TCGTGGAAGGACTCATGACC | CCCGTTCAGCTCAGGGATG |
| **18S** | CGATCAGATACCGTCGTAGTTC | GGTCATGGGAATAACGCCGC |
| **28S** | GAAGCGCGGGTAAACGGC | TGACGAGGCATTTGGCTACC |
| **5S** | CATACCACCCTGAACGCGCC | CCGACCCTGCTTAGCTTCCG |

**Table S2. Fold enrichment of DPC signal across chromatin states**

| State | C1 | C2 | C3 | T1 | T2 | T3 | R1 | R2 | R3 |
| --- | --- | --- | --- | --- | --- | --- | --- | --- | --- |
| Active TSS | 0.94797 | 0.47506 | 0.5663 | 0.56905 | 0.34968 | 0.36062 | 0.19929 | 0.15449 | 0.11977 |
| Flanking TSS | 0.96321 | 0.70278 | 0.75218 | 1.15282 | 1.05505 | 0.92969 | 0.80592 | 0.59419 | 0.62809 |
| Flanking TSS Upstream | 1.04542 | 0.79951 | 0.86314 | 0.84381 | 0.53916 | 0.66548 | 0.32373 | 0.23546 | 0.16038 |
| Flanking TSS Downstream | 1.05454 | 0.86546 | 0.90298 | 1.07033 | 0.75904 | 0.84612 | 0.47343 | 0.30707 | 0.23068 |
| Strong Transcription | 1.05836 | 1.03291 | 1.05954 | 1.44331 | 1.22389 | 1.28289 | 0.37114 | 0.30849 | 0.25498 |
| Weak transcription | 1.05297 | 1.05895 | 1.07065 | 1.19114 | 1.07539 | 1.13767 | 0.65637 | 0.58243 | 0.52711 |
| Genic Enhancer 1 | 1.05461 | 0.9502 | 1.01799 | 1.49224 | 1.08186 | 1.18178 | 0.25061 | 0.18181 | 0.12865 |
| Genic Enhancer 2 | 1.06994 | 0.95211 | 1.03586 | 1.22502 | 0.74518 | 0.85382 | 0.15713 | 0.08345 | 0.06287 |
| Active Enhancer 1 | 1.0876 | 1.08195 | 1.10904 | 1.02289 | 0.77244 | 0.93832 | 0.64359 | 0.52651 | 0.43779 |
| Active Enhancer 2 | 1.05169 | 1.05482 | 1.05384 | 0.99133 | 0.93952 | 1.03921 | 0.77194 | 0.80796 | 0.75626 |
| Weak Enhancer | 1.08255 | 1.04731 | 1.07387 | 1.50454 | 1.23925 | 1.3066 | 1.06451 | 0.76545 | 0.7358 |
| ZNF genes & repeats | 1.02118 | 0.89469 | 0.93914 | 1.65394 | 1.86619 | 1.66103 | 1.83795 | 1.43732 | 1.89563 |
| Heterochromatin | 1.01052 | 0.99363 | 0.98881 | 0.95025 | 1.08364 | 1.05291 | 1.21103 | 1.30534 | 1.31821 |
| Bivalent/poised TSS | 0.97021 | 0.40975 | 0.52185 | 2.65535 | 2.5783 | 2.06172 | 2.24711 | 1.38337 | 1.68977 |
| Bivalent Enhancer | 1.07028 | 0.58297 | 0.74419 | 3.5632 | 3.28395 | 2.78419 | 3.264 | 1.65222 | 2.38582 |
| Repressed PolyComb | 1.06859 | 0.7786 | 0.87057 | 3.45944 | 3.33495 | 2.68489 | 3.58254 | 1.86131 | 3.26374 |
| Weak Represses PolyComb | 1.06262 | 0.89618 | 0.96253 | 2.59806 | 2.43873 | 2.09596 | 2.85166 | 1.70062 | 2.51203 |
| Quiescent/Low | 0.98267 | 1.00929 | 0.99858 | 0.81131 | 0.84448 | 0.86766 | 0.89532 | 1.01347 | 0.94372 |

**Table S3. Total number of mapped reads in Q0, Q4, and rDNA genes after filtering.**

| **Genes** | **Replicate** | **Total number of reads after filtering** | | **p value**  **(between T0 and T5)** |
| --- | --- | --- | --- | --- |
|  |  | **T0** | **T5** |  |
| **Q0 genes** | 1 | 859590 | 962821 | 0.49 |
|  | 2 | 846941 | 823885 |  |
|  | 3 | 952559 | 875556 |  |
| **Q4 genes** | 1 | 1302070 | 909591 | 0.004 |
|  | 2 | 1218654 | 662628 |  |
|  | 3 | 1433438 | 944277 |  |
| **rDNA genes** | 1 | 21261 | 21184 | 0.13 |
|  | 2 | 18522 | 18109 |  |
|  | 3 | 18701 | 16992 |  |
